# *Tamarix aphylla* derived metabolites ameliorate indomethacin-induced gastric ulcers in rats by modulating the MAPK signaling pathway, alleviating oxidative stress and inflammation: *In vivo* study supported by pharmacological network analysis

**DOI:** 10.1371/journal.pone.0302015

**Published:** 2024-05-10

**Authors:** Faisal H. Altemani, Abeer H. Elmaidomy, Dalia H. Abu-Baih, Azza M. Abdel Zaher, Fatma Alzahraa Mokhtar, Naseh A. Algehainy, Hussain T. Bakhsh, Gerhard Bringmann, Usama Ramadan Abdelmohsen, Omnia Hesham Abdelhafez

**Affiliations:** 1 Department of Medical Laboratory Technology, Faculty of Applied Medical Sciences, University of Tabuk, Tabuk, Saudi Arabia; 2 Department of Pharmacognosy, Faculty of Pharmacy, Beni-Suef University, Beni-Suef, Egypt; 3 Department of Biochemistry & Molecular Biology, Faculty of Pharmacy, Deraya University, Minia, Egypt; 4 Deraya Center for Scientific Research, Deraya University, Minia, Egypt; 5 Department of Pathology, Faculty of Medicine, Minia University, Minia, Egypt; 6 Fujairah Research Centre, Fujairah, UAE; 7 Department of Pharmacy Practice, Faculty of Pharmacy, King Abdulaziz University, Jeddah, Saudi Arabia; 8 Institute of Organic Chemistry, University of Würzburg, Würzburg, Germany; 9 Department of Pharmacognosy, Faculty of Pharmacy, Minia University, Minia, Egypt; 10 Department of Pharmacognosy, Faculty of Pharmacy, Deraya University, Minia, Egypt; American University of Madaba, JORDAN

## Abstract

Nature has proven to be a treasure resource of bioactive metabolites. In this regard, *Tamarix aphylla* (F. Tamaricaceae) leaves crude extract was investigated for its gastroprotective effect against indomethacin-induced damage to the gastric mucosa. Additionally, phytochemical investigation of the methanolic extract afforded eight flavonoids’ derivatives (**1–8**). On pharmacology networking study, the isolated compounds identified 123 unique targets where only 45 targets were related to peptic ulcer conditions, these 45 targets include 11 targets specifically correlate to gastric ulcer. The protein-protein interaction defined the PTGS2 gene as one of the highly interacted genes and the complete pharmacology network defined the PTGS2 gene as the most represented gene. The top KEGG signaling pathways according to fold enrichment analysis was the EGFR tyrosine kinase inhibitor resistance pathway. As a result, these findings highlighted the significance of using *T*. *aphylla* leaves crude extract as an anti-gastric ulcer candidate, which provides a safer option to chemical antisecretory medicines, which are infamous for their negative side effects. Our findings have illuminated the potent anti-inflammatory and antioxidant effects of *T*. *aphylla*, which are likely mediated by suppressing IL-1β, IL-6, TNF-α, and MAPK signaling pathways, without compromising gastric acidity.

## 1 Introduction

The occurrence of gastric ulcer, a widely observed gastrointestinal disorder, impacts a substantial proportion of the worldwide population, with prevalence rates ranging from 0.71% to 1.41% [1, 2]. Multiple factors contribute to its emergence, including smoking, anxiety, alcohol consumption, *Helicobacter pylori* (*H*. *pylori*) infection, and nonsteroidal anti-inflammatory drugs (NSAIDs). Curiously, NSAIDs are responsible for a quarter of all gastric ulcers [3–5], among them, indomethacin, a potent NSAID used to treat severe inflammation conditions, garners attention. Nonetheless, the biggest impediment of the drug is its propensity to induce ulceration within the gastric mucosa. If this matter is not duly addressed, it may give rise to grave complications, including gastric bleeding and perforation [6]. The potent ulcerogenic effect of indomethacin on the gastric mucosa has made it a go-to choice in laboratory research for inducing gastric ulcers in diverse in *vivo* models [7]. Multiple explanations have been proposed to elucidate the mechanism behind the development of gastric ulcers caused by indomethacin. These hypotheses include the suppression of cyclooxygenase activity, disruption of the antioxidant system in the gastric mucosa, and imbalances in the secretion of crucial components like prostaglandins, bicarbonate and mucous, elicitation of gastric mucosal inflammation, and provocation of apoptosis [3–6].

The primary approach to shield NSAID users from peptic ulcer and mucosal injury is by using proton pump inhibitors (PPIs) prophylactically. Nevertheless, there is mounting evidence that the use of PPIs can worsen the intestinal damage caused by NSAIDs by altering the composition of intestinal microbiota [8] and heightening the risk of gastric cancer [9]. Consequently, it is imperative to explore complementary and alternative medicine to tackle NSAIDs-induced gastric injury. A whole series of studies have demonstrated the efficacy of natural agents in mitigating NSAID-induced gastric ulcerations. This can be attributed to their robust antioxidant and anti-inflammatory characteristics, without detrimental consequences [10]. Recent research conducted by Al-Awaida et al. (2023) [11] has shed light on the potential benefits of Epigallocatechin Gallate (EGCG) in protecting against the harmful effects of water-pipe smoke. This study suggests that natural compounds may have the ability to counteract cellular damage caused by inflammation and oxidative stress. In a fascinating study, researchers explored the effects of wheatgrass on breast cancer cells in a simulated microgravity environment [12]. Their findings shed light on the potential benefits of plant-derived substances in managing disease conditions by influencing gene expression and improving histopathological outcomes. Another study investigated the impact of green tea consumption on antioxidant and inflammation-related genes, further highlighting the potential of plant-based compounds in promoting health [13].

The MAPK signaling pathway consists of three axes: extracellular signal-regulated kinase (ERK), c-Jun N-terminal kinase (JNK), and p38 [14]. In earlier research, it was established that the MAPK signaling pathway holds significant sway over the control of gastrointestinal damage caused by NSAIDs, exerting a pivotal influence on inflammatory responses [15, 16], apoptosis [17], and wound healing [18].

Over years, natural product-derived medicine has been known as a resource for the treatment of different diseases [19–21]. Herbal medications have revealed a potent contribution in drugs manufacturing to cure different ailments [22–25]. Nowadays, available drugs are metabolites, combinations, in addition to dilutions obtained from nature [26–29]. On the other hand, one of the most fruitful plant families are the Tamaricaceae, which comprises nearly 80 rheophytes, halophytes, as well as xerophytes growing in semi-arid as well as in dry regions, particularly in central and southwest Asia [30–32].

Additionally, the genus *Tamarix* L., commonly known as tamarisk, comprises more than 60 species of different plants, which are utilized by local people in Asia and Africa for treatment of various medicinal diseases as infections, wounds, liver in addition to spleen ailments [32, 33]. Leaves of these plants are distinguished with their needle-like appearance, which are enclosed by salt, obtained from their salt glands [31, 34]. Moreover, *T*. *aphylla* (synoyms *T*. *articulata*, *T*. *orientalis*, *Thuja aphylla*, *Tetraclinis aphylla*) is a perennial tree distributed in different regions of the world such as Asia, Central Africa, and Middle East. Additionally, it was reported that the plant is utilized in folk medicine to treat several illnesses such as dental infections, leprosy, and colds. It has also been used as carminative, antimicrobial, anti-oxidant, aphrodisiac, anthelmintic, diuretic, anti-hemorrhoid, anti-inflammatory, and antidiarrheal medicine, and against skin diseases, Ulcer, and GIT disorders [35]. The leaves have been utilized in folk medicine to cure wounds, abscesses, and rheumatism. Different investigations have revealed that *T*. *aphylla* showed various bioactivities as an antipyretic, analgesic, anti-rheumatic, as well as anti-inflammatory [36–38]. On the other hand, *T*. *aphylla* leaves are rich in valuable bioactive metabolites, such as steroids, flavonoids, terpenoids, cardiac glycosides, tannins, polyphenols, and essential oils [35, 36, 39, 40], while their stem bark are valuable source of polyphenols, saponins, coumarins, flavonoids, tannins, triterpenes, and alkaloids [41, 42].

Flavonoids are a varied class of polyphenolic compounds with different scaffolds obtained mainly from medicinal plants [43, 44]. They are responsible for the yellow color of these plants [45]. The bioactivities of many plants are attributed to their flavonoids, which showed a remarkable spectrum of activities, such as cardioprotective [46], neuroprotective [47], anti-inflammatory, antispasmodic [48], anticholinesterase, anti-oxidant, and anticancer [49–51]. Clinical investigations have revealed the potency of numerous plants for the management of gastroduodenal disorders [52, 53]. However, till now *T*. *aphylla* has not garnered substantial interest in terms of unearthing its hidden treasures. Recent scientific investigations have unveiled the latent potential harbored within this botanical specimen, which necessitates further exploration and elucidation. Accordingly, our current research sought to investigate the efficacy of *T*. *aphylla* in protecting against indomethacin-induced gastric ulcers, while unveiling the underlying potential mechanisms, with particular emphasis on its role in the MAPK pathway, oxidative stress and inflammation, and its ability to mitigate histopathological alterations in the stomach. Moreover, isolation of different flavonoids in addition to a pharmacological network analysis were employed to discern the chemical compounds that contribute to the anti-ulcer activity.

## 2 Materials and methods

*T*. *aphylla* leaves were gathered from the desert of El-Minya Government. The plant was kindly identified by Dr. Abd ElHalim A. Mohammed (Department of Flora and Phytotaxonomy Research, Dokki, Cairo, Egypt). A voucher specimen (2022-BuPD 91) has been deposited at the Department of Pharmacognosy, Faculty of Pharmacy, Beni-Suef University, Egypt.

### 2.1. Extraction of *T*. *aphylla* leaves and fractionation

*T*. *aphylla* leaves (2.0 kg) were collected, air-dried in the shade for one month, then powdered utilizing an OC-60B/60B grinding machine (60–120 mesh, Henan, Mainland China). Additionally, the powdered leaves were macerated in 70% methanol (3 × 7 L, 7 d each) at room temperature, as well as concentrated under vacuum at 45°C utilizing a rotary evaporator (Buchi Rotavapor R-300, Cole-Parmer, Vernon Hills, IL, USA) affording 300 g crude extract. The dried extract was suspended in 150 mL distilled water (H_2_O) as well as partitioned with various solvents according to their polarities (*n*-hexane, DCM, EtOAc, and *n*-butanol). For every step, the organic phase was separated and concentrated under vacuum at 45°C utilizing a rotary evaporator (Buchi Rotavapor R-300, Cole-Parmer, Vernon Hills, IL, USA) affording the following fractions I (10.0 g), II (30.0 g), III (5.0 g) and IV (8.0 g), respectively. Moreover, the remaining mother liquor was concentrated affording the aqueous fraction (V). The afforded fractions were stored at 4°C for biological and phytochemical experiments [23, 54].

### 2.2. Isolation and purification of compounds

Fraction IV (8.0 g) was fractionated using VLC on a silica gel column (6 × 30 cm, 50 g). It was eluted utilizing EtOAc: acetic acid: formic acid: H_2_O 10: 0.1: 0.1: 2 isocratic mixture (4 L each). The effluents were gathered in fractions, concentrated followed by monitoring by TLC utilizing the system EtOAc: acetic acid: formic acid: H_2_O 10: 1: 1.2 and PAA reagent. Accordingly, similar fractions were gathered and concentrated providing four sub-fractions: IV_1_-V_4_. Sub-fractions IV_1_ and IV_2_ were then purified separately on a Sephadex^®^ LH-20 column (0.25–0.1 mm, 100 × 0.5 cm, 100 g) eluted using MeOH affording compounds **1** (30 mg), **2** (20 mg), and **6** (10 mg). Sub-fractions IV_3_ and IV_4_ were additionally purified separately, on C‐_18_ RP‐HPLC utilizing H_2_O‐CH_3_CN (10–60%, 30 min, 5 mL/min), affording compounds **3** (8 mg), **4** (5 mg), **5** (12 mg), **7** (7 mg), and **8** (10 mg).

### 2.3. *In vitro* antioxidant assay

#### 2.3.1. Hydrogen peroxide scavenging activity

A particular amount of externally sourced hydrogen peroxide was employed to investigate the effectiveness of the antioxidant capacity [55]. By using a colorimetric approach, the residual hydrogen peroxide was identified. The colored by-product intensity at 505 nm permitted to determine the percentage of inhibition, which reflected the overall scavenging activity of the sample in comparison to the blank. Vitamin C was used as a standard reference using the equation:

$$Scavenging\;activity=\frac{A\;control-A\;sample}{A\;control}\times 100$$

The GraphPad Prism 8 program was used to calculate the IC_50_ using four distinct concentrations.

#### 2.3.2. Superoxide radical scavenging activity

The methodology of Sreenivasan and colleagues was meticulously followed to quantify the superoxide anion scavenging potential [56]. The optical density was determined at 560 nm and compared with that of the standard, ascorbic acid. Finally, the % inhibition was estimated by the equation:

$$Superoxide\;scavenging\;activity=\frac{A\;control-A\;sample}{A\;control}\times 100$$

Using four different concentrations, IC_50_ was estimated by using the GraphPad Prism 8 program.

### 2.4. Network pharmacology and gene ontology analysis

#### 2.4.1. (Plant–cpds) network

The phytochemical investigation of *T*. *aphylla* was performed resulting in the identification of eight compounds which are kaempferol and quercetin related derivatives, a basic network linking the plant *T*. *aphylla* to the identified compounds was constructed.

#### 2.4.2. (Cpds–target) networks

The Cpds—target network was assembled utilizing chemical data acquired for each compound from the PubChem database (https://pubchem.ncbi.nlm.nih.gov/) [57] (last accessed on 18-08-2023) and SwissTargetPrediction Database (http://www.swisstargetprediction.ch/result.php?job=215444691&organism=Homo_sapiens) [58] (last accessed on 23-8-2023) was utilized to determine targets associated with each compound in relation to the Homo sapiens species. Specifically, the SwissTargetPrediction Database was utilized to select the top targets, ensuring a probability score greater than 0.

#### 2.4.3. (Target—peptic ulcer) network

DisGenet (https://www.disgenet.org/) [59] (last accessed on 27-8-2023) online database was utilized to ascertain the target genes associated with peptic ulcer conditions. The targets–gastric ulcer conditions were filtered using keywords; ‘‘gastric ulcer”, ‘‘peptic ulcer”, ‘‘duodenal ulcer” and ‘‘ulcerative colitis”

#### 2.4.4. Protein-protein interaction (PPI) network

A network was constructed between the targets related to gastric ulcer only to find out the interactions between the gastric ulcer targets, the PPI network was constructed through the STRING database [60] (last accessed on 29-9-2023), with no application of more or less functions. with confidence score ≥ 0.4

#### 2.4.5. Complete pharmacology network

Plant-cpd-target network was formed by combining the (Plant—cpd, Cpd—gastric ulcer target, Gastric ulcer target-gastric ulcer). This network and previously formed networks were constructed, visualized, and analyzed using the software Cytoscape 3.9.0. (https://cytoscape.org/download.html) [61].

#### 2.4.6. Gene ontology and enrichment analysis

The gene ontology and enrichment analysis were conducted on all the genes of the compounds being studied that are involved in peptic ulcer. This analysis aimed to identify the GO terms related to cellular components, molecular function, and biological processes that are influenced by the annotated genes using the ShinyGO 0.75 database (http://bioinformatics.sdstate.edu/go/) (a graphical gene set enrichment tool) [62] (accessed on 28-8-2023).

### 2.5. *In vivo* gastric ulcer model

#### 2.5.1. Animals

The adult male Wistar rats (weighing between 180 and 200 grams) were procured from the animal house of the Faculty of Pharmacy at Deraya University in Minia, Egypt. In order to mitigate animal distress, the rats were given a 1-week acclimation period before the start of the experiment, and all necessary precautions were taken to ensure their comfort. The study adhered to ethical guidelines and regulations set by the experimental animal center and research ethics committee, Deraya University, Minia, Egypt (Ethical approval number 17/2022) and in compliance with the ARRIVE guidelines [63]. The rats were housed in five separate cages. To maintain normal laboratory conditions, the rats were kept at a temperature of 22°C, with a humidity level ranging between 50–55%. Additionally, a 12-hour light/dark cycle was implemented to regulate their daily rhythms. They were provided with ad libitum access to standard food and water and all measures to reduce animal suffering were followed. To avoid any influence of diurnal rhythms on gastric functions, all animals were employed in the study at an identical time of day [64]. To increase the study’s repeatability and guarantee that any reported effects or outcomes are related to the specified treatments, we created clear and consistent selection criteria including:

- Only male rats were used to reduce sex hormone differences.
- Rats of a certain age and weight were chosen to reduce variance due to growth and metabolism.
- Rats were randomly assigned to groups to achieve fair distribution of individual traits and reduce confounding effects.
- Uniform dosage, frequency, and duration for indomethacin, famotidine, or *T*. *aphylla* leaf extract.

#### 2.5.2. Experimental design

**Fig 1:**
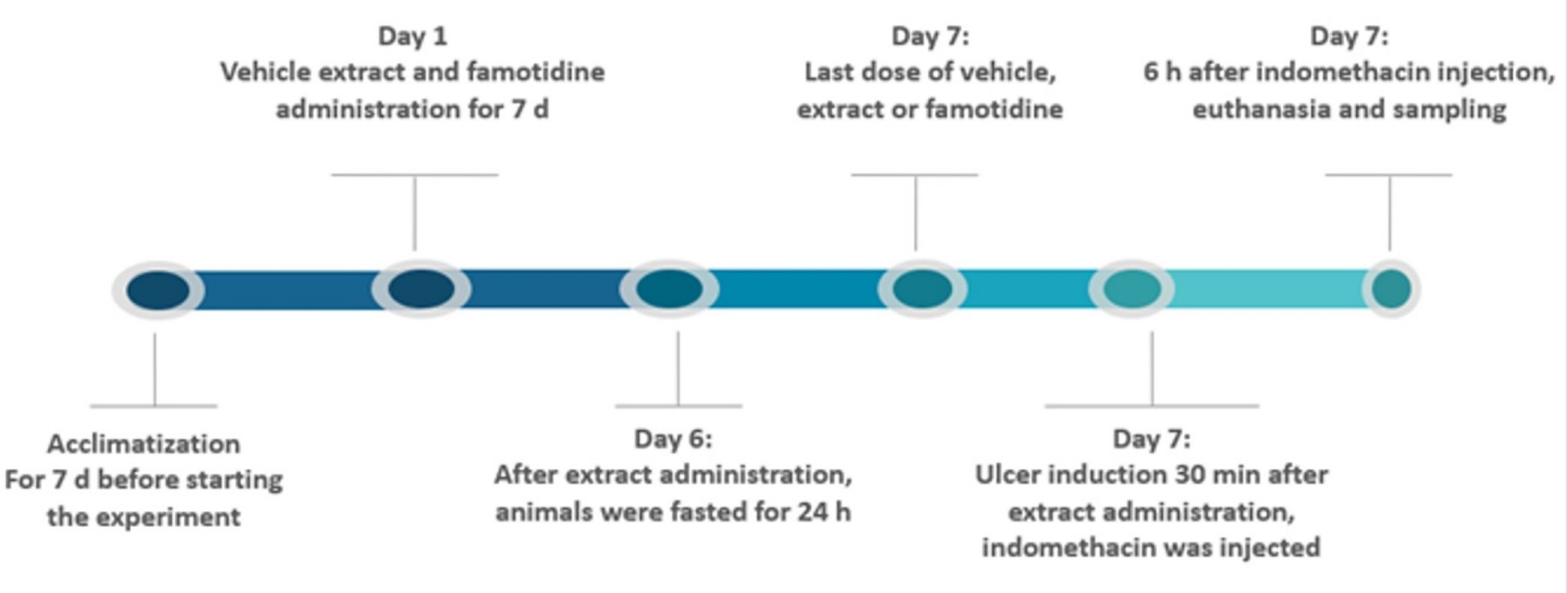
Time course of the experimental schedule.

Twenty-four rats were artfully split into four groups, with six rats per group, each group being subjected to different oral treatments. The first group was given a vehicle consisting of 0.5% carboxy methyl cellulose (CMC) for a week (1 mL). The second group was treated with the same vehicle for a week, followed by indomethacin on the seventh day only. The third group received oral famotidine 10 mg/kg suspended in 1 mL of 0.5% CMC for 7 d and indomethacin on day 7. The fourth group got 250 mg/kg *T*. *aphylla* leaf extract for seven days and indomethacin on day seven as illustrated in Fig 1. The dose of *T*. *aphylla* was selected based on previous literature [65, 66] The gastric lesions were performed using an adapted Djahanguiri method [67]. After fasting for 16 h with free water, the animals received 40 mg/kg indomethacin orally [68].

#### 2.5.3. Blood and tissue sampling

6 hours after receiving indomethacin, animals were carefully anesthetized using thiopental sodium at a dose of 50 mg/kg [69] and sacrificed by decapitation. The stomachs were delicately extracted for additional experimental procedures, and meticulously dissected along the larger curvature to acquire the gastric contents. A portion of the gastric region from each rat was carefully removed, weighed, and preserved in a solution of 10% formalin-saline for further histopathological investigations. The remaining portion was quickly frozen using liquid nitrogen and stored at a temperature of -80°C for future tissue analyses.

#### 2.5.4. Assessment of gross mucosal damage

The gastric region of every rat was meticulously rinsed with saline and delicately dried using a pair of filter papers. The stomachs, after undergoing purification, were delicately elongated and affixed onto a corkboard. Subsequently, digital images were obtained to conduct a comprehensive evaluation of mucosal damage. The acquired images were thoroughly examined utilizing ImageJ software, (Wayne Rasband, MD, USA). Subsequently, the ulcerated regions were accurately quantified to ascertain the ulceration ratio, as described by Szabo and Hollander [70]. In order to determine the ulcer index (U.I.) for each rat, the subsequent equation was utilized:

$$U.I.=\frac{ulcerated\;area}{Total\;stomach\;area}\times 100$$

Finally, the percentage of inhibition against ulceration was computed using this formula:

$$\%\;ulcer\;inhibition=\frac{\;U.I.in\;ulcer\;group-U.I.in\;test\;group}{\;U.I.in\;ulcer\;group}\times 100$$

#### 2.5.5. Microscopic examination of gastric ulcers

The stomach tissues were fixed in a 10% solution of neutral buffered formalin for 24 hours. Afterwards, they went through a process of dehydration and were then embedded in paraffin wax. After that, the tissues were cut into thin sections measuring 5 μm using a sledge microtome. Hematoxylin and eosin (H&E) staining was used to facilitate histopathological analysis with the light microscope [71].

#### 2.5.6. Measurement of gastric acidity

In accordance with the methodology outlined by Beiranvand et al., the pH of the gastric juice was assessed. Each rat’s stomach contents were centrifuged for 10 minutes at 5000 rpm, yielding a supernatant that was then blended with 1 milliliter of distilled water. Next, a pH metre (Fisherbrand, AB315 tabletop pH metre, Waltham, MA, USA) was used to test the pH [7, 72].

#### 2.5.7. Assessment of gastric tissue oxidative stress markers

To assess the oxidative stress and antioxidant activity in the stomach, the gastric homogenate was used to evaluate the level of malondialdehyde (MDA) and reduced glutathione (GSH) level. The measurements were performed using colorimetric methods described by Tappel and Zalkin [73] for MDA and by Ellman [74] for GSH. The results are expressed as nmol/g tissue for MDA and mmol/g for GSH.

#### 2.5.8. Gene expression analysis

*Total RNA extraction*. A stomach tissue sample weighing 50 mg was homogenized using an ultrasonic homogenizer (Sonics-Vibracell, Sonics and Materials Inc., USA) in 0.5 mL of TRIzol reagent (Invitrogen, Thermo-Fisher, Amresco, LLC-Solon, USA) [75]. The manufacturer’s protocol was meticulously adhered to in order to extract total RNA from the gastric tissues. In order to ascertain the integrity of the RNA, precise measurements and estimations were conducted to evaluate both the concentration and purity of the RNA [76].

*Real-time PCR (qRT-PCR)*. To get a better understanding of gene expression, we utilized the Revert Aid H Minus First Strand cDNA Synthesis kit from Thermo Scientific. This kit was used to reverse transcribe equal RNA conc in all samples as directed by the manufacturer. Maxima SYBER Green kit was used for qRT-PCR. The primers were carefully chosen for their specificity and reliability. For qRT-PCR, 2 μg of RNA per reaction was used, and 40 cycles of amplification. The reaction was carried out using the StepOne™ Real-Time PCR Detection System from Applied Biosystems. To account for variations in gene expression, the data underwent normalization with respect to the housekeeping gene, *GAPDH*. The relative RNA abundances were calculated using the comparative Ct approach, 2 ^(-ΔΔCt)^ [77].

#### 2.5.9. Determination of gastric tissue protein expression of the MAPK pathway using western blotting assay

The Bradford method was then employed to calculate protein concentrations [78]. 40 μg of total proteins were resolved for direct immunoblotting, we employed SDS-PAGE for a duration of 1 hour at a voltage of 100 volts. The proteins were subsequently relocated to PVDF membranes. Following membrane blocking, the membranes were subjected to the addition of primary antibodies: rabbit anti-Erk1/2 (catalogue number: AF0155, Affinity bioscience), rabbit anti-p-Erk1/2 (catalogue number: 9102, Cell Signaling Technology, USA), rabbit anti-p38 (catalogue number: 9212, cell signaling, USA), rabbit anti- p-p38 (catalogue number: 9211, cell signaling technology, USA), rabbit anti-JNK (catalogue number: E-AB-31853, Elabscience, USA), rabbit anti-p-JNK (catalogue number: AP0631, ABclonal, USA), and GAPDH (catalogue number: M01263) (Boster Biological Technology, Pleasanton, CA, USA). After that, the membranes underwent overnight inoculation at 4° C. HRP secondary antibody (Cell Signaling Technology Inc., Massachusetts, USA) was used. Bands were visible by chemiluminescence by an enhanced chemiluminescence kit (ECL, GE Healthcare, Chicago, IL, USA) and identified using an image analyzer for luminescent data (LAS-4000, Fujifilm Co., Tokyo, Japan). Following normalization to GAPDH, the densitometric analysis of protein bands from different groups was performed, and their intensities were compared to the untreated group using ImageJ Software.

### 2.6. Statistical analysis

Results are presented as mean ± SD. Tukey’s post hoc test was employed following a one-way ANOVA to adjust for multiple comparisons using GraphPad Prism 8 (GraphPad Software Inc., La Jolla, CA, USA).

## 3. Results

### 3.1. Hydrogen peroxide scavenging

**Fig 2:**
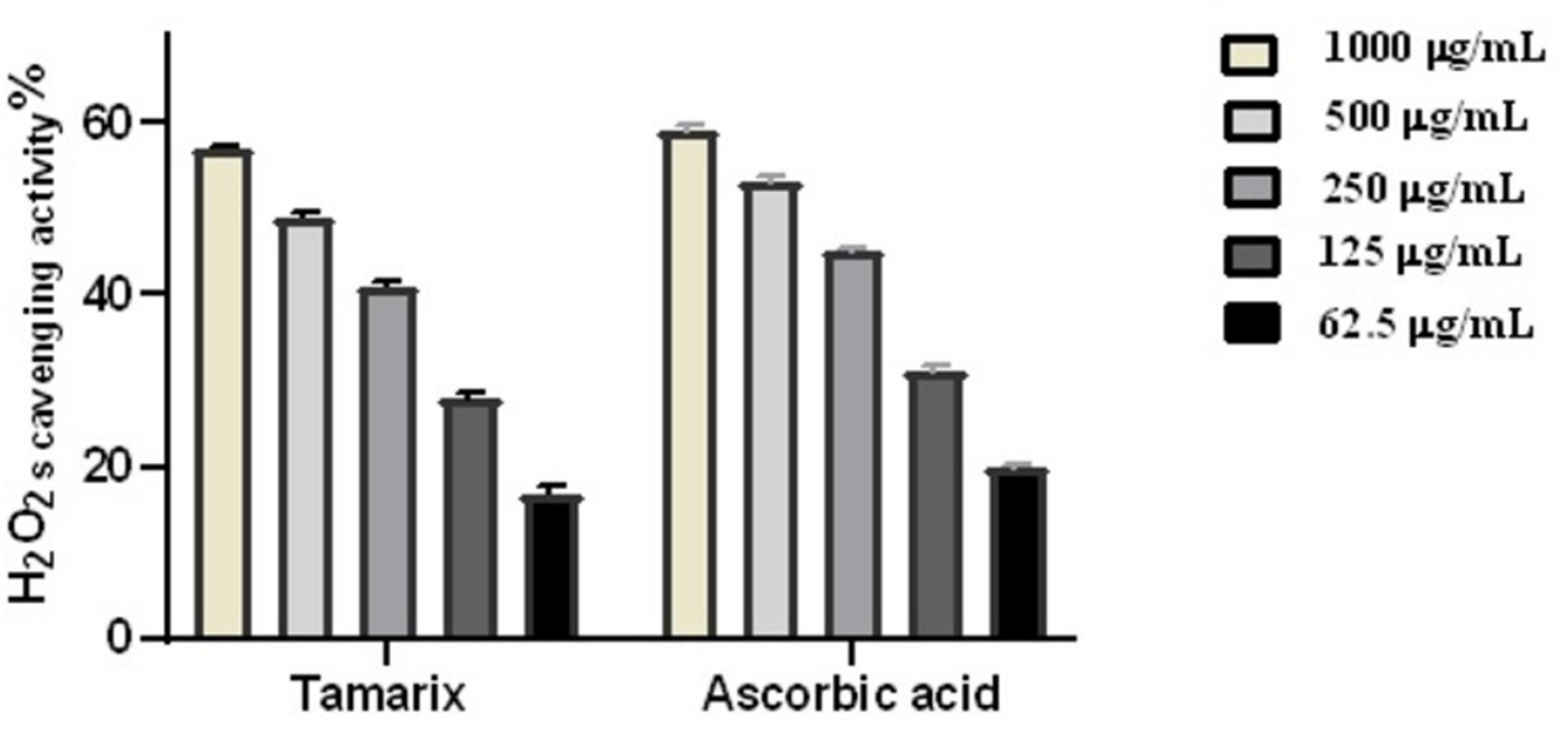
*T*. *aphylla* crude extract’s ability to scavenge H_2_O_2_ radicals at various doses (1000 μg/mL, 500 μg/mL, 250 μg/mL, 125 μg/mL, and 62.5 μg/mL). The bars display mean ± SD. A one-way ANOVA test was employed to analyze any significant differences among the groups.

In this study, the ability of *T*. *aphylla* crude leaf extract to scavenge H_2_O_2_ and to act as an antioxidant was evaluated. The results showed that at 1000 g/mL of H_2_O_2_, *T*. *aphylla* exhibited a maximum 57% H_2_O_2_ radical scavenging activity. With an IC_50_ of 123.4 g/mL compared to 108.1 g/mL for ascorbic acid (the positive control), *T*. *aphylla* crude leaf extract notably and concentration-dependently reduced the formation of H_2_O_2_-related radicals, suggesting consistent antioxidant capability (Fig 2).

### 3.2. Superoxide radical scavenging

**Fig 3:**
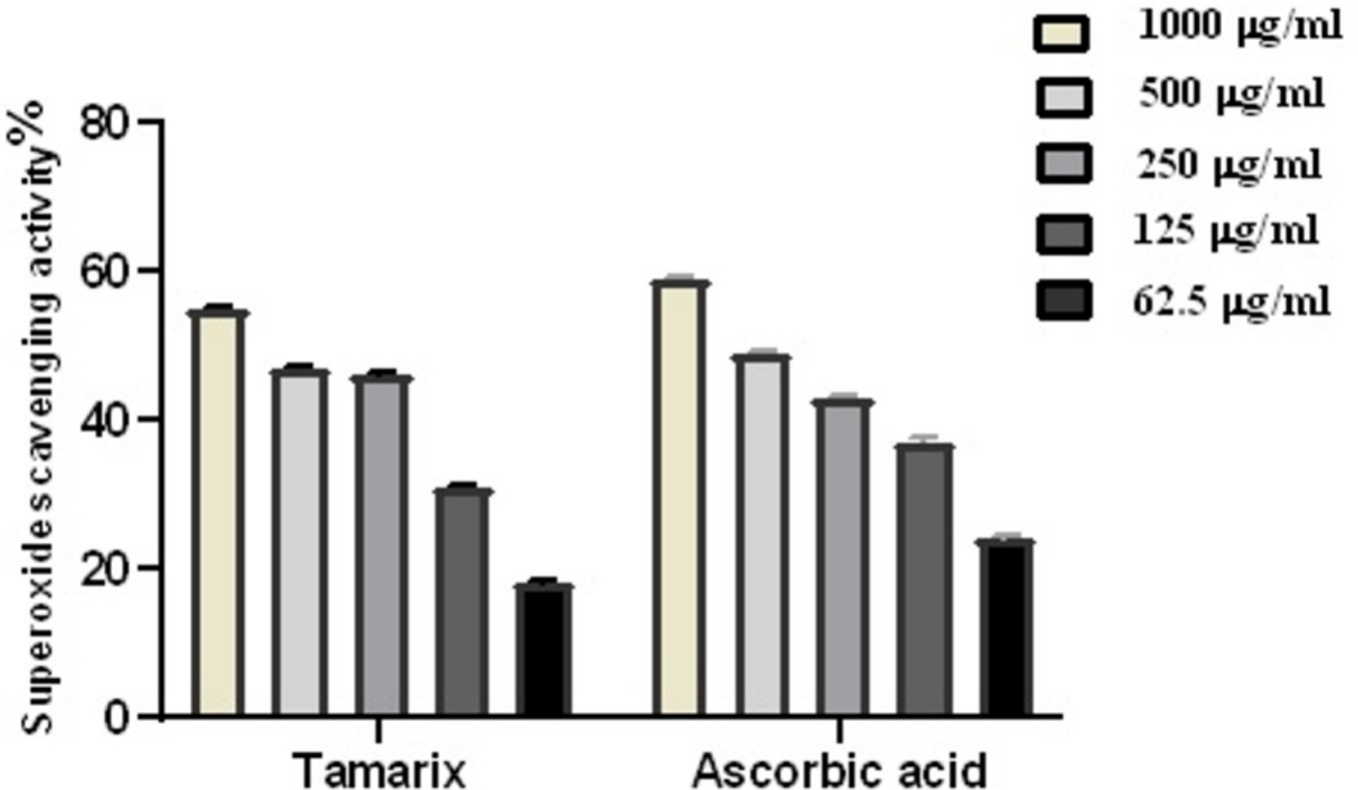
*T*. *aphylla* crude leaf extract’s superoxide scavenging activity at different doses (1000 μg/mL, 500 μg/mL, 250 μg/mL, and 125 μg/mL, 62.5 μg/mL). The bars show the mean ± SD. A one-way ANOVA test is used to assess significant differences among groups.

The ability of *T*. *aphylla* crude leaf extract to neutralize superoxide was examined. The results showed that *T*. *aphylla* crude extract considerably and dose-dependently decreased superoxide radicals, showing a consistent antioxidant capacity with an IC_50_ of 103.8 g/mL in contrast to ascorbic acid (IC_50_ = 86.72 g/mL) Fig 3.

### 3.3. Isolation of secondary metabolites from *T*. *aphylla* crude leaf extract:

**Fig 4:**
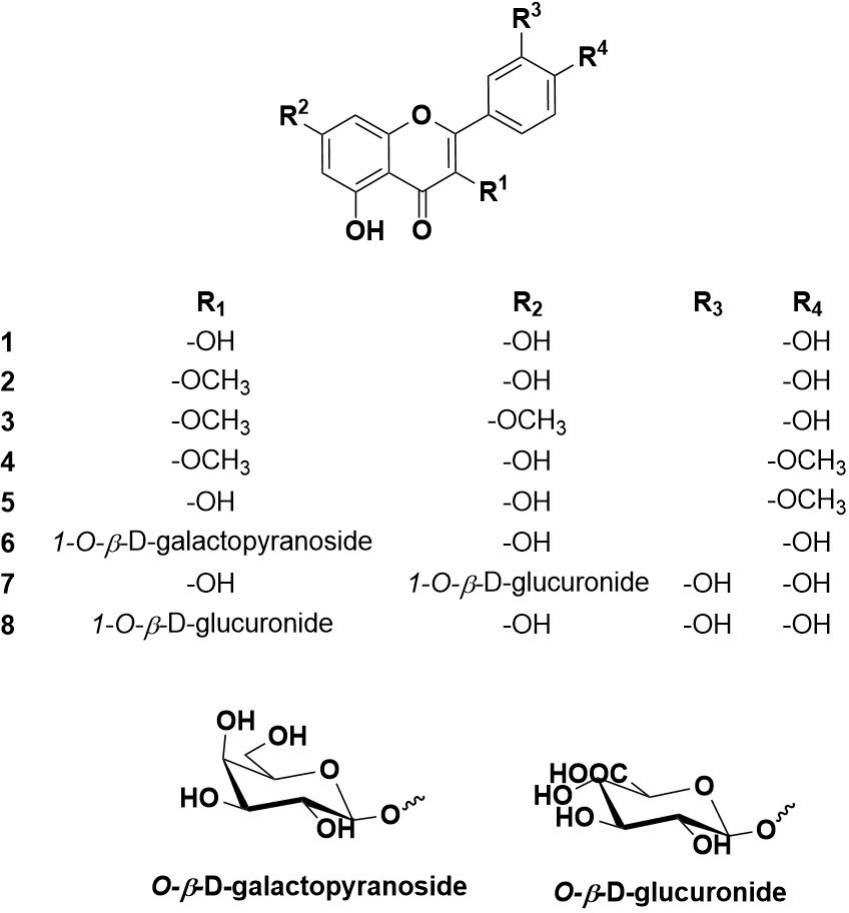
The isolated flavonoids from *T*. *aphylla* leaves.

Based on the physicochemical and chromatographic properties, the spectral analyses from UV, ^1^H, and DEPT-Q NMR, as well as comparisons with the literature and some authentic samples, the crude methanolic extract of *T*. *aphylla* leaves afforded the known compounds, kaempferol (**1**) [79], 3-methylkaempferol (**2**) [80], kumatakenin (**3**) [81], ermanin (**4**) [82], kaempferide (**5**) [81], trifolin (**6**) [83], quercetin 7-*O-β*-D-glucuronide (**7**) [84], quercetin 3-*O-β*-D-glucuronide (**8**) [84] (Figs 4 and S1–S11).

### 3.4. (Plant–cpd) network

*T*. *aphylla* afforded eight compounds including kaempferol aglycone and seven derivatives of kaempferol and quercetin. The identified compounds were illustrated in a simple network called (Plant—cpd) network (S12 Fig).

### 3.5. (Cpd–target) network

The eight identified compounds were correlated to their targets through SwissTargetPrediction database, the total identified targets were 123 unique targets, a simple network correlated the identified targets to the compound was constructed (S13 Fig). The formed cpd-target network consisted of 131 nodes and 347 edges, the nodes represent the compounds and corresponding identified targets.

### 3.6. (Target—peptic ulcer) network

**Fig 5:**
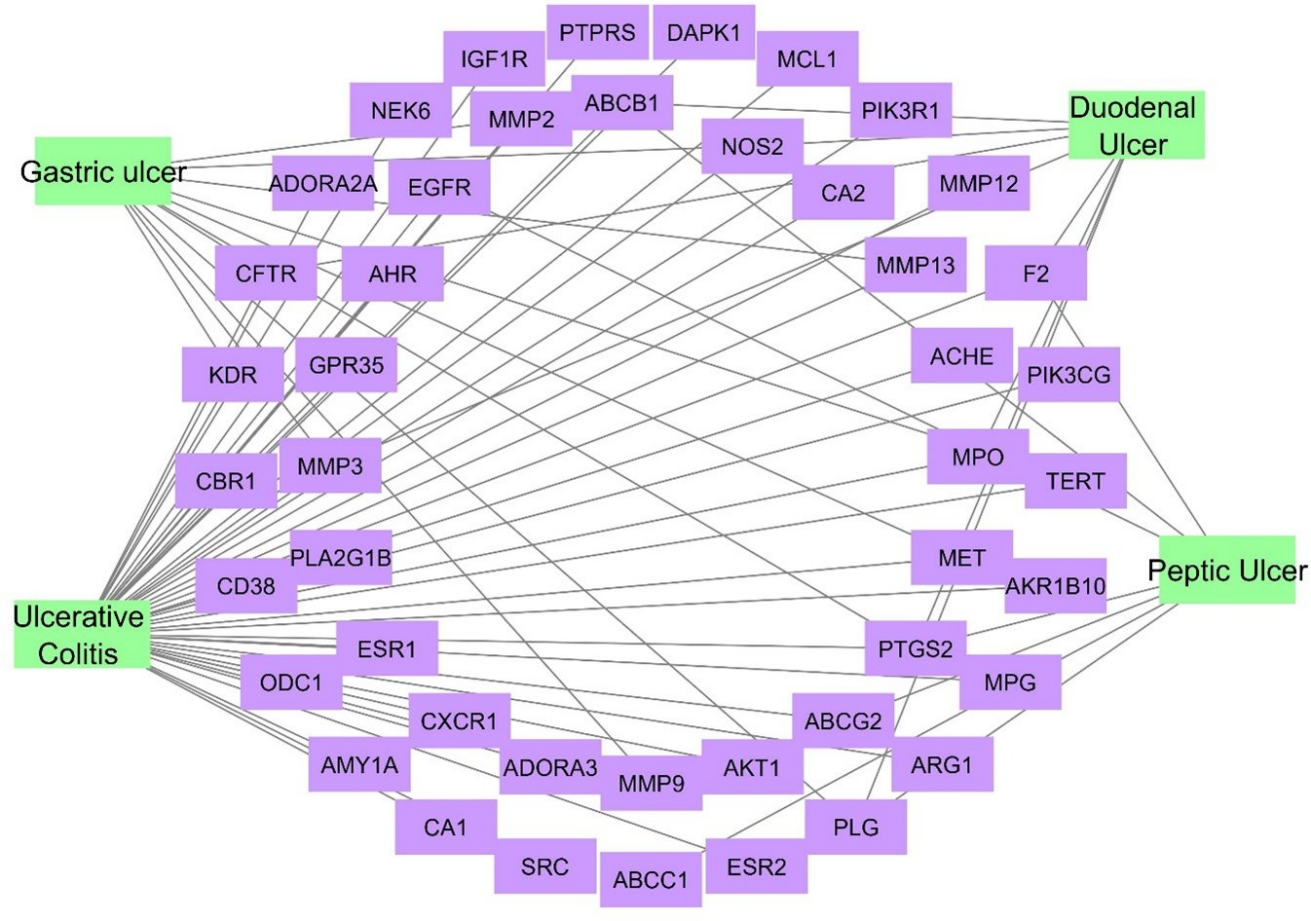
A; Target-peptic ulcer network, a network connects the targets to different peptic ulcer conditions where the violet rectangles represent the targets related to peptic ulcer conditions and green rectangles represent different peptic ulcer conditions.

Through DisGeNET database a total number of 35062 gene-disease associations were obtained for the target genes, upon filtration to focus on target-peptic ulcer conditions; only 45 targets were related to peptic ulcer conditions, the formed network consisted of 49 nodes and 69 edges (Fig 5). The gastric ulcer was affected by 11 targets only, the gastric ulcer targets are PTGS2, NOS2, MMP13, CFTR, MMP3, MMP2, MET, PLG, KDR, MMP9 and TERT (S14 Fig).

### 3.7. Reversed network

A reversed network Gastric ulcer-target and gastric ulcer targets-cpds networks was constructed to connect the gastric ulcer targets to the identified compounds to determine the compounds that correlate to gastric ulcer and other compounds not related to gastric ulcer condition (S15 Fig). Kaempferol was found to be linked to all the 11 targets of peptic ulcer.

### 3.8. Protein-protein interaction (PPI)

**Fig 6:**
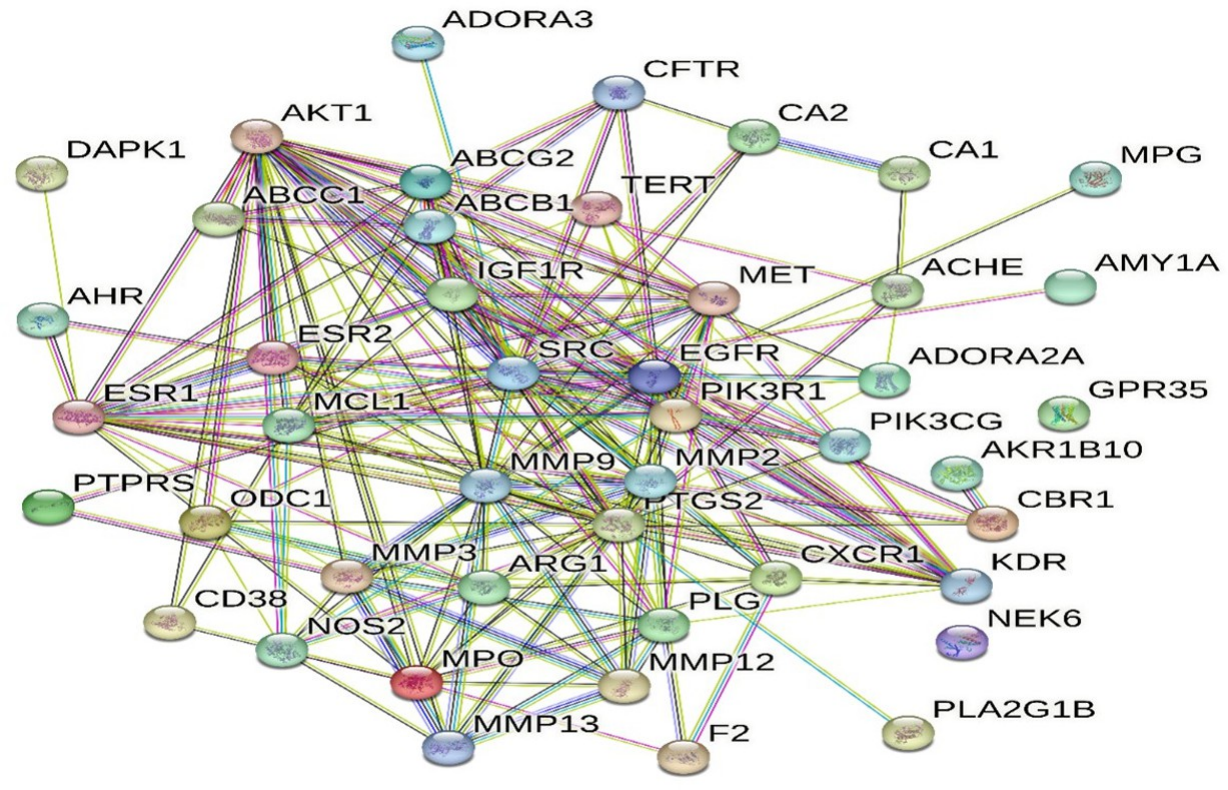
Protein-protein interactions of targets identified by *T*. *aphylla* compounds.

The PPI network was constructed for the peptic ulcer genes, the constructed network to show the interactions between peptic ulcer targets proved that the following genes have the higher node degrees; AKT1, TNF, SRC, EGFR, ESR1, PTGS2, MMP9 (Fig 6).

### 3.9. Complete network pharmacology

**Fig 7:**
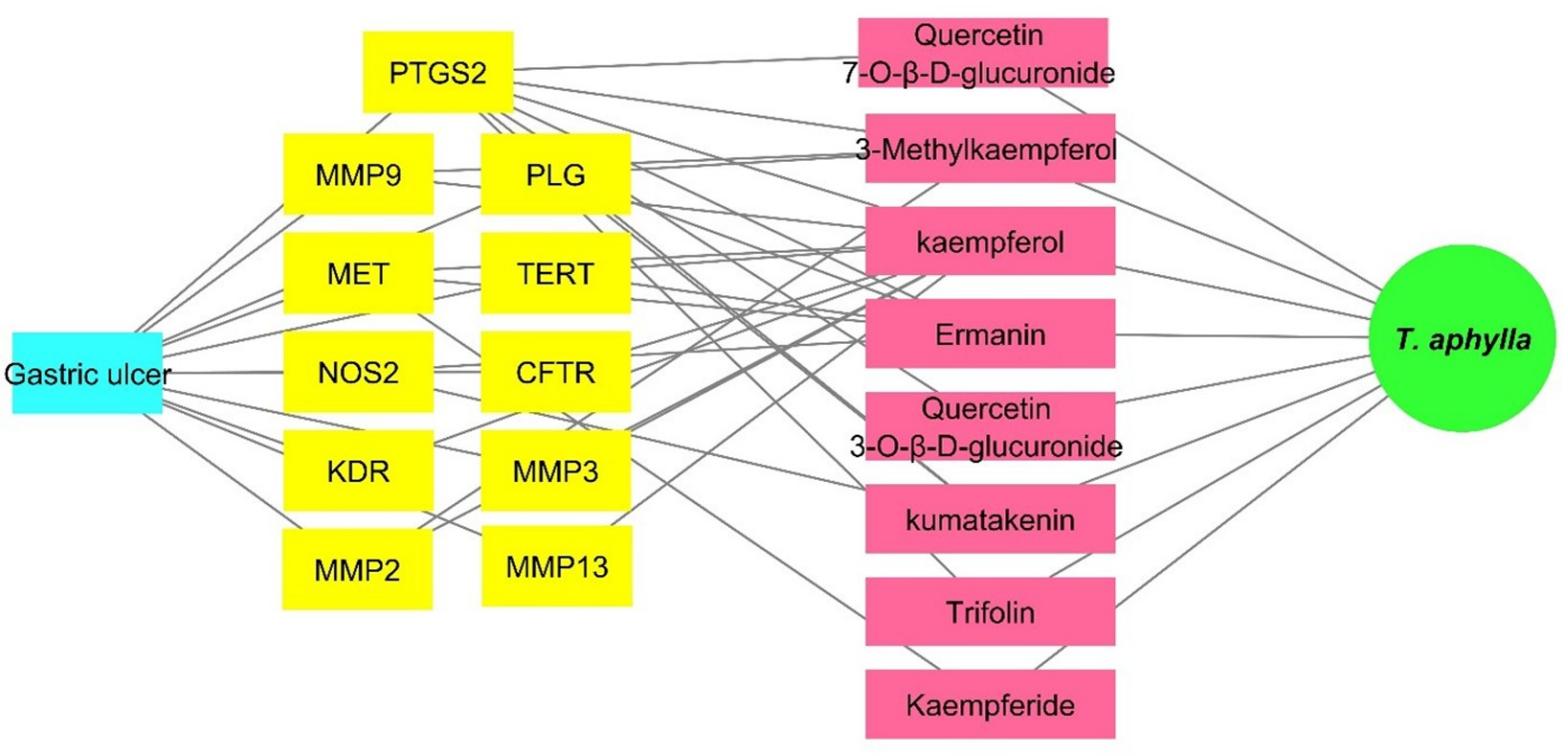
A complete pharmacology network: A complete pharmacology network connect the plant *T*. *aphylla* (in green circle) to the identified compounds (pink rectangles), and the targets (yellow rectangles) to the gastric ulcer (blue rectangle).

By combining the previously constructed networks (Plant-cpd., Cpd–gastric ulcer target, Gastric ulcer target—gastric ulcer) in a merged network; a complete pharmacology network was evolved (Fig 7). This network consisted of 21 nodes and 44 edges (Fig 7). From the complete pharmacology network, PTGS2 was found to be the top gene with seven edges related to seven identified compounds.

### 3.10. Gene ontology (GO) and gene enrichment analysis

**Fig 8:**
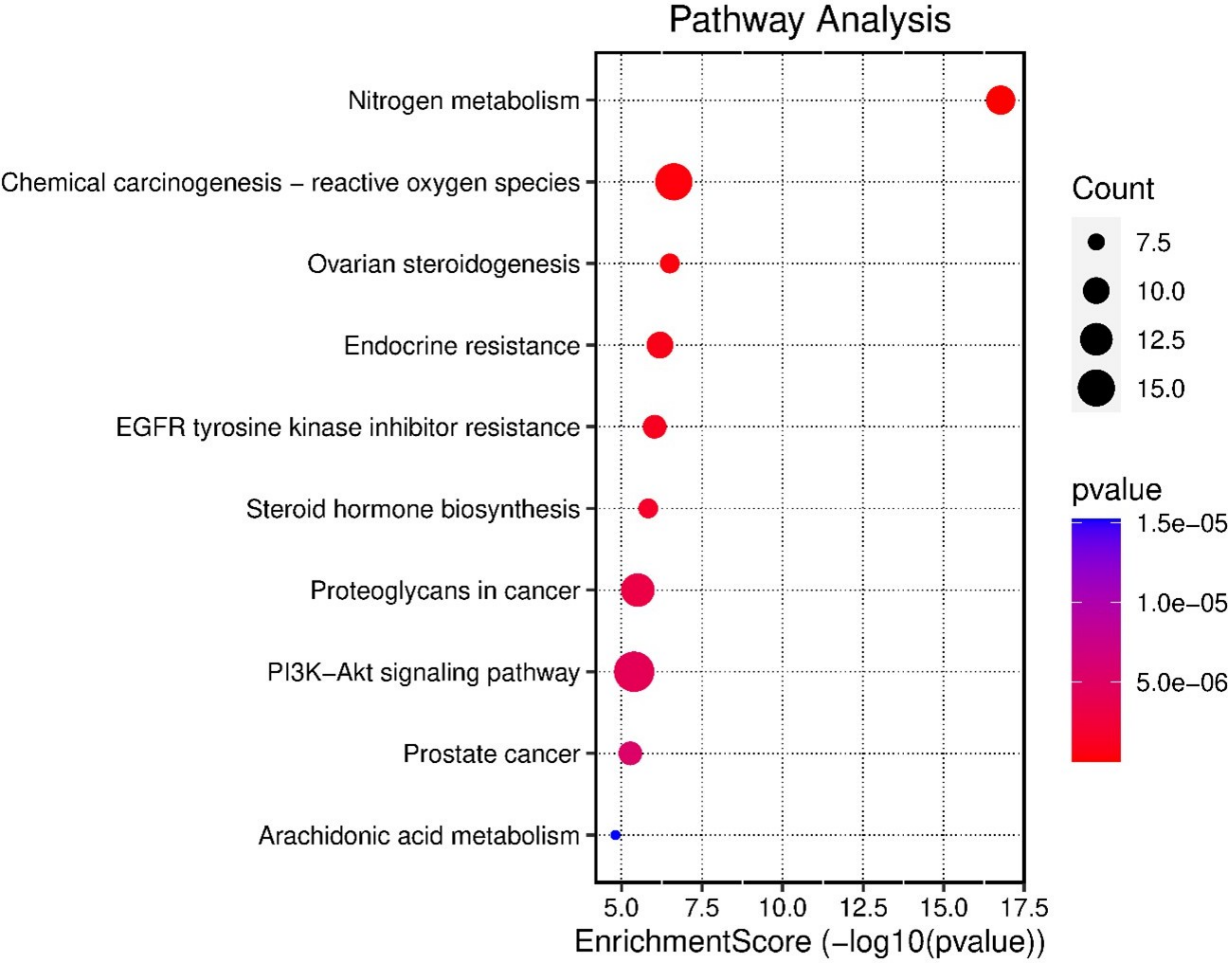
Chart of the top 10 KEGG pathways arranged according to enrichment score.

**Fig 9:**
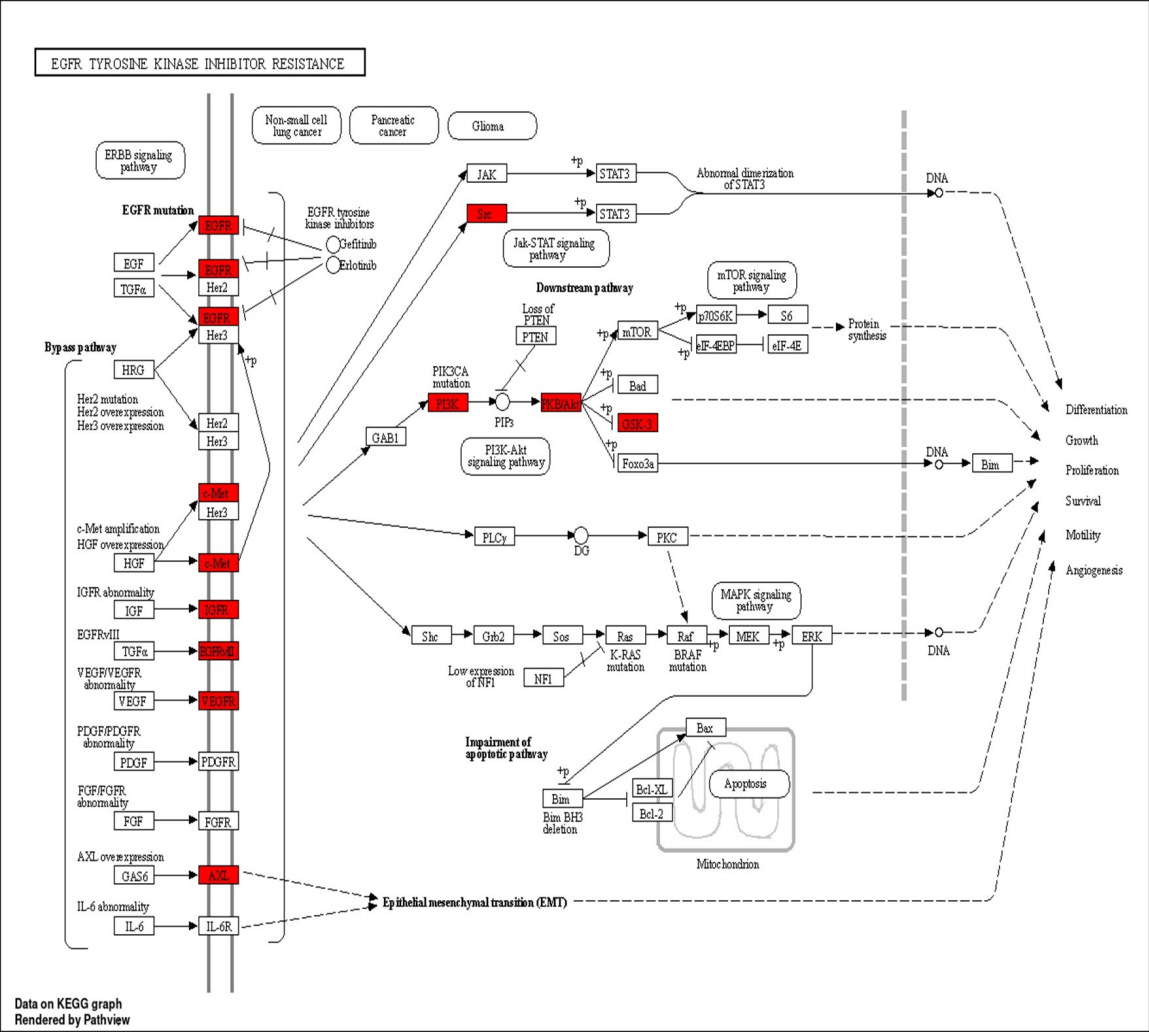
The top biological signaling pathway. Red colored genes are the identified genes in the pathway.

The gene enrichment analysis was performed to the 12 targets found to be involved in gastric ulcer treatment, the top biological process is bicarbonate transport (GO:0015701), cellular response to chemical stress (GO:0062197) and response to oxidative stress (GO:0006979) (S16 Fig and S1 Table). The top cellular component is apical part of cell (GO:0045177), membrane raft (GO:0045121), and membrane microdomain (GO:0098857) (S16 Fig). The top molecular function is carbonate dehydratase activity (GO:0004089), protein serine/threonine kinase activity (GO:0004674), and hydro-lyase activity (GO:0016836) (S16 Fig). The top KEGG signaling pathways according to fold enrichment was EGFR tyrosine kinase inhibitor resistance (Fig 8). The tyrosine kinase inhibitor resistance signaling pathway possesses its activities by different pathway cascades including MAPK signaling pathway, PI3K-AKT signaling pathway and mTOR signaling pathway (Fig 9).

### 3.11. Effect of *T*. *aphylla* leaf on UI of indomethacin-treated animals

**Fig 10:**
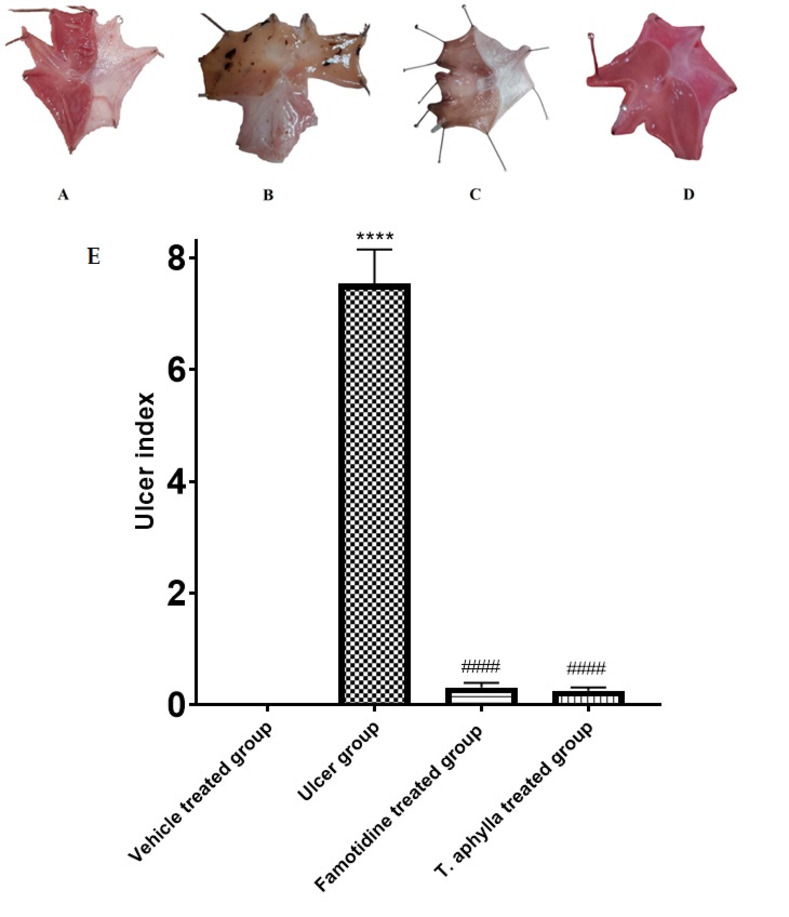
Photomacrographs of the rat stomach. (A) a control group, (B) indomethacin, (C) famotidine, and (D) *T*. *aphylla*. The indomethacin group exhibited a notable presence of both round and linear gastric ulcers. However, the administration of famotidine and *T*. *aphylla* extract resulted in a substantial inhibition of these ulcers. (E) The impact of indomethacin, alone and in conjunction with pre-administrations of famotidine, or *T*. *aphylla* extract, on the ulcer index. The statistical assessments were performed employing ANOVA and Tukey’s post hoc test. The sample size was n = 6, and the data are displayed as the mean ± SD. **** The observed data show a substantial difference compared to the control group, with a statistically substantial level of *p* < 0.0001. #### The observed results also show a significant difference relative to the rats administered indomethacin alone (ulcer group), with a statistical significance level of *p* < 0.0001.

Upon macroscopic examination, it was observed that the stomachs obtained from the control rats displayed an absence of apparent inflammation or ulceration. Furthermore, the mucosa exhibited its typical healthy pink coloration, along with its characteristic folding and thickness. The stomachs of the rats treated with indomethacin were isolated, revealing hemorrhagic longitudinally aberrant mucosal lesions of various sizes and depths. However, the examination of stomachs extracted from the groups treated with *T*. *aphylla* crude leaf extract or famotidine displayed minor mucosal lesions and congestion. These findings indicate that both the reference medication (famotidine) and the extract exhibited preventive properties. Comparing to the control rats, the administration of indomethacin significantly increased the UI (7.5) (*p* < 0.0001). However, the crude extract of *T*. *aphylla* exhibited a significant reduction in UI (0.25, *p* < 0.0001) relative to the group treated with indomethacin. These findings were parallel to the results observed with famotidine. It is noteworthy to mention that there was no statistically substantial disparity observed among the control group and the groups administered famotidine or *T*. *aphylla* (Fig 10).

### 3.12. Histopathological study

**Fig 11:**
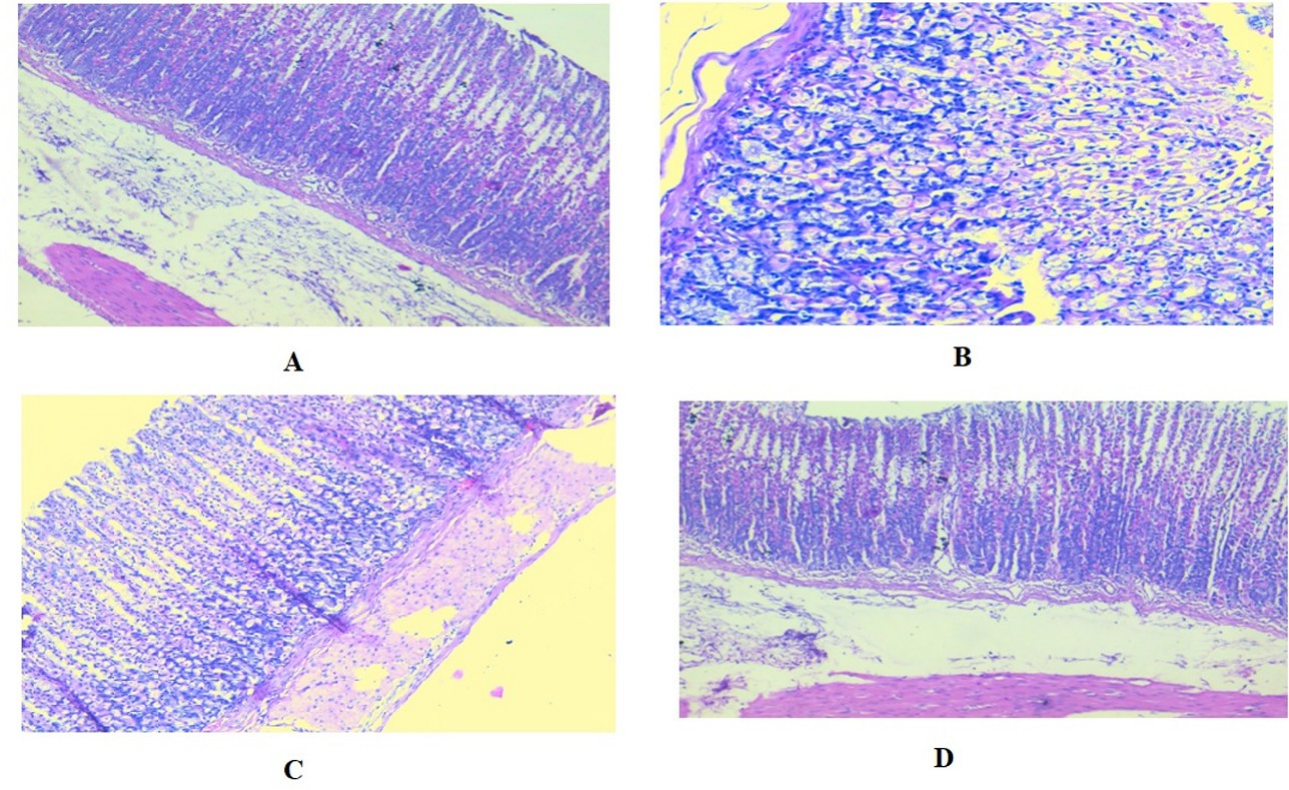
A: Section of unaltered gastric mucosa displaying typical surface mucous cells and regular parietal cells. B: the experimental group treated with indomethacin, a distinct region displaying extensive coagulative necrosis can be observed in the gastric mucosa. (C): famotidine treated group, a distinct reduction in the degenerative and necrotic alterations within the gastric mucosa was observed. (D): group treated with the crude leaf extract of *T*. *aphylla*, a thorough examination of the section displayed a remarkable recovery from gastric ulcer, characterized by the restoration of the normal mucosa and the absence of coagulative necrosis. (H&E × 100 & 200).

Histopathological analysis of the gastric specimens from various rat groups unveiled a conspicuous manifestation of gastric impairment subsequent to the administration of indomethacin. This was substantiated by the presence of pronounced coagulative necrotic alterations in the gastric mucosa, concomitant with the infiltration of inflammatory cells. Famotidine pre-administration significantly reduced the observed pathological alterations in the stomachs, as revealed by a notable decrease in degenerative and necrotic changes within the gastric mucosa. The administration of the extract to the rats prior to indomethacin treatment exhibited a notable decrease in the degenerative alterations observed in the gastric glands. This reduction was attained by effectively inhibiting the occurrence of inflammation in the mucosal lining (Fig 11).

### 3.13. Effects of *T*. *aphylla* leaf extract on gastric acidity in indomethacin treated rat

**Fig 12:**
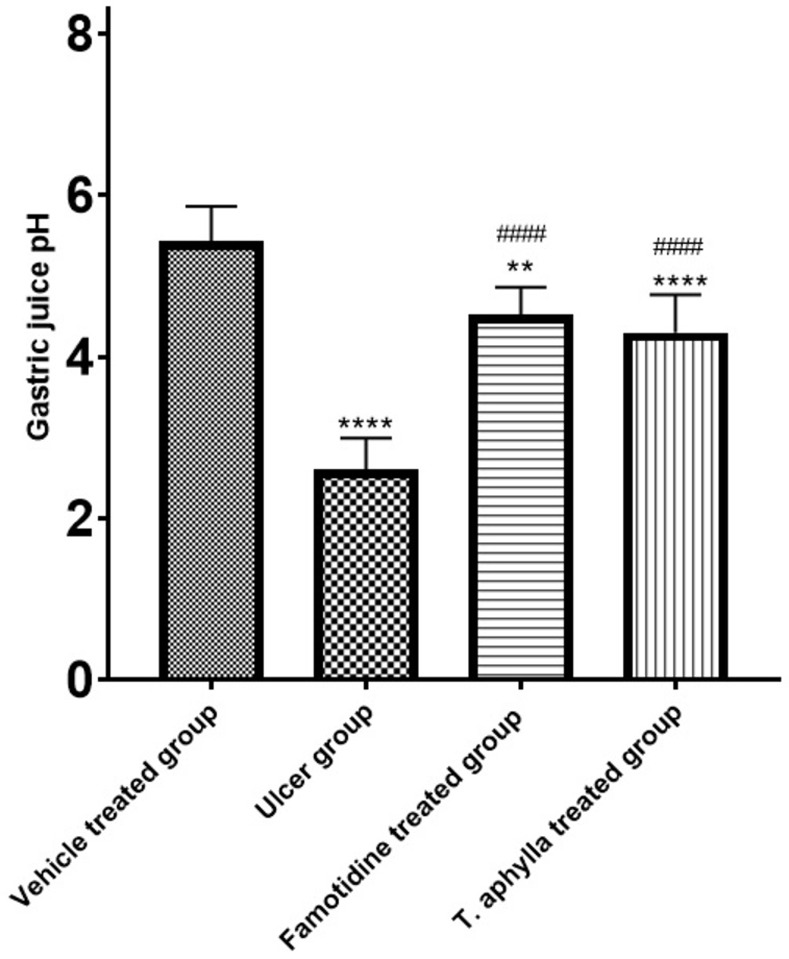
The impact of oral administration of indomethacin alone or in combination with famotidine or *T*. *aphylla* extract on the gastric pH. Statistical assessments were done utilizing ANOVA and Tukey’s post hoc test. The sample size was 6, and the data are displayed as the mean ± SD. **** *p* < 0.0001, ** *p* < 0.01 as compared to the control group. #### *p* < 0.0001 as compared to ulcer group.

The study revealed a noticeable surge in gastric acidity demonstrated by a substantial reduction in gastric pH (reaching 2.6, *p* < 0.0001) in rats subjected to indomethacin treatment in comparison with control rats (Fig 12). The administration of either famotidine or the crude extract from *T*. *aphylla* leaded to a notable reduction in gastric acidity, as evidenced by pH values of 4.5 and 4.3, respectively (*p* < 0.0001). This reduction was observed when compared to the indomethacin group. Notably, there was no discernible difference in the effectiveness of famotidine and the *T*. *aphylla* crude extract in reducing gastric acidity, as indicated in Fig 12.

### 3.14. The impact of *T*. *aphylla* leaf on gastric MDA and GSH levels upon indomethacin administration

**Fig 13:**
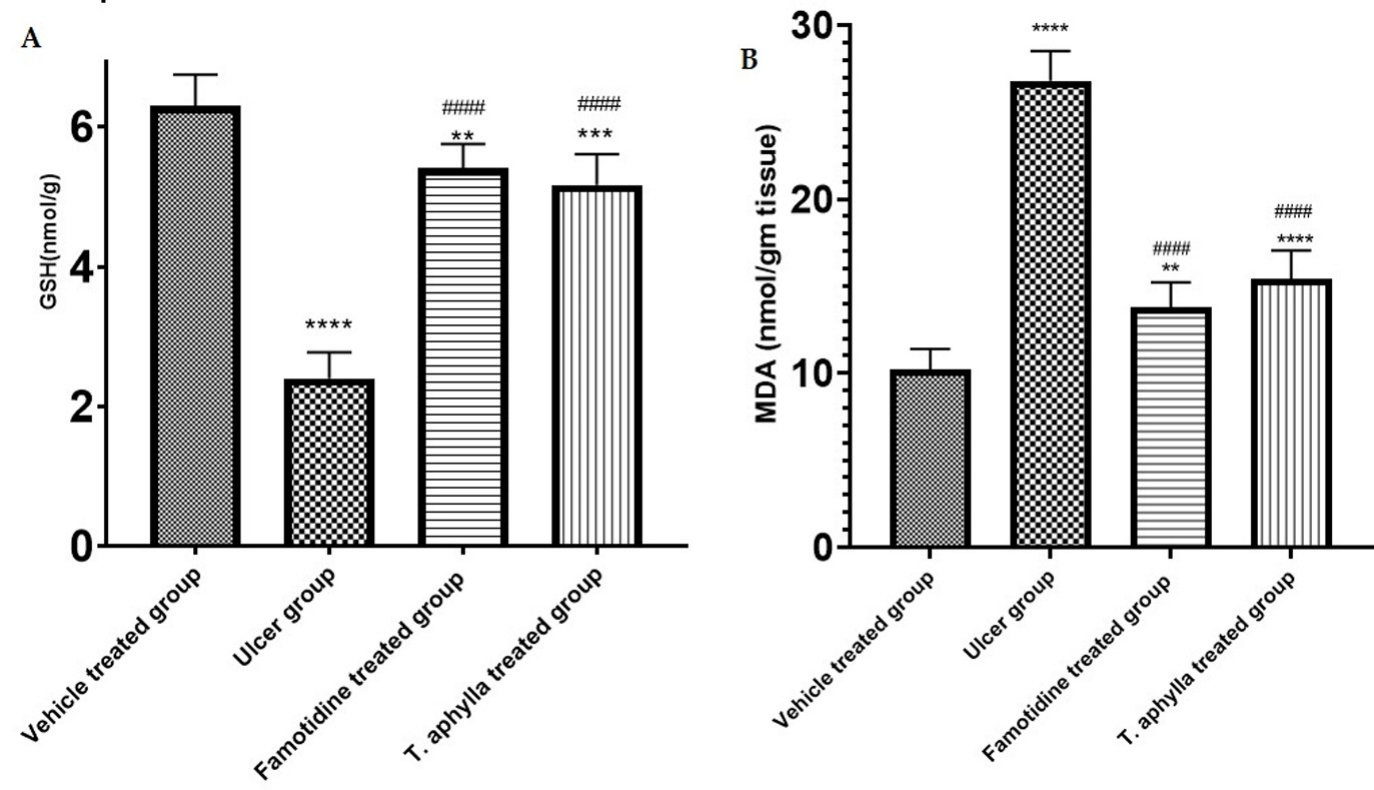
The impact of indomethacin on (A) stomach GSH and (B) MDA, both alone and in conjunction with oral famotidine or *T*. *aphylla* extract administration. ANOVA was utilized for statistical assessment and tukey’s post hoc test. The mean ±SD is employed to show the data, n = 6. ** *p* < 0.01. ***, *p* < 0.001, ****, *p* < 0.0001 as compared to the control group. #### *p* < 0.0001.

In order to unravel the protective potential of *T*. *aphylla*, we delved deeper into the analysis of oxidative stress biomarkers within gastric mucosa. Impressively, indomethacin was observed to reduce gastric GSH by a staggering degree to 2.4 mmol/g tissue in comparison with the control rats (*p* < 0.0001) (Fig 13). Famotidine or *T*. *aphylla* leaf pre-administration, however, elevated the GSH levels by a substantial amount to 5.4 and 5.16 mmol/g tissue individually (*p* < 0.0001) (Fig 13), elucidating the remarkable protective effect of both the extract and famotidine. Conversely, indomethacin appeared to significantly increase MDA level in stomach tissues in comparison with the control rats (*p* < 0.0001). The administration of famotidine or the extract, however, substantially reduced MDA production to a substantial amount of 13.8 and 15.4 nmol/g tissue correspondingly (*p* < 0.0001) (Fig 13), exhibiting the potential of these agents to combat oxidative damage in the mucosa of the stomach.

### 3.15. Effect of *T*. *aphylla* on mRNA level of proinflammatory cytokines

**Fig 14:**
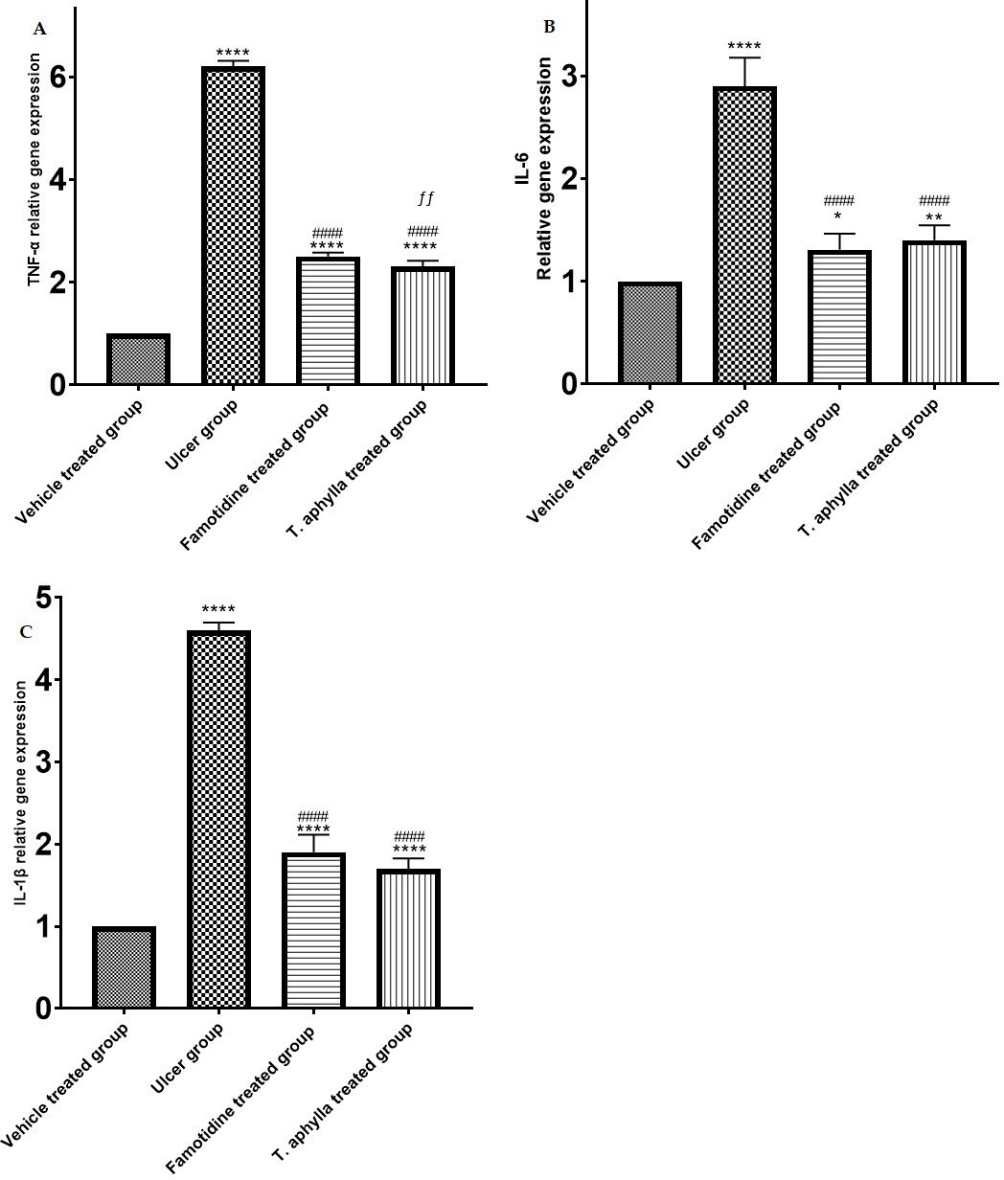
mRNA levels of the proinflammatory cytokines TNF-α, IL-6, and IL-1β in all groups. The fold change values were reported relative to the control group following normalization with respect to GAPDH. The bars represent the mean ± SD. The ANOVA test was employed to assess the significant divergence among the groups. * *p* < 0.05, ** *p* < 0.01, **** *p* < 0.0001 as compared to control group. #### when comparing to ulcer rats *p* < 0.0001. ƒƒ *p* < 0.01 as compared to the famotidine group.

The injection of indomethacin resulted in a notable inflammatory reaction in the gastric region, as indicated by the considerable increases in IL-6 (2.86-fold), TNF-α (6.2-fold) and IL-Iβ (4.58-fold) relative to the control rats (*p* < 0.0001). Notably, the administration of *T*. *aphylla* leaf extract exhibited a remarkable reduction in the heightened concentrations of IL-6 (1.38-fold), TNF-α (2.27-fold) and IL-1β (1.67-fold), demonstrating statistical significance relative to rats administered indomethacin (*p* < 0.0001). Additionally, it is worth noting that famotidine exhibited a comparable impact by substantially reducing the levels of inflammatory indicators, specifically IL-6 (1.26-fold), TNF-α (2.46-fold) and IL-1β (1.86-fold), relative to the indomethacin rats (*p* < 0.0001) (as illustrated in Fig 14).

### 3.16. Effect of *T*. *aphylla* leaf extract on the MAPK pathway

**Fig 15:**
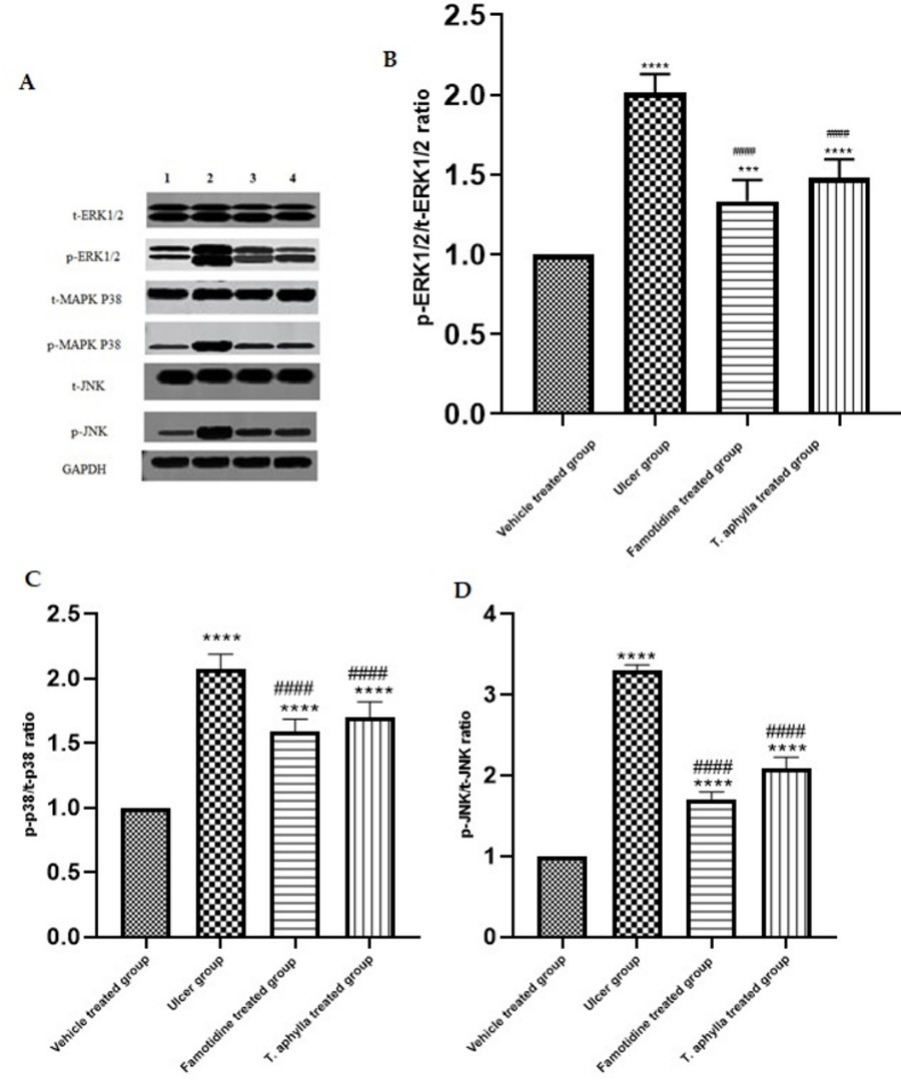
A: Representative immune blots of t-ERK1/2, t-MAPK P38, t-JNK, p-ERK1/2, p-MAPK P38, and p-JNK in all the study groups. (B-D): The quantification of phosphor-ERK1/2/total ERK1/2, phosphor-P38/total P38, and phosphor-JNK/total JNK was determined by normalizing the expression levels to GAPDH. The data were presented as fold change relative to the control group. Densitometric analysis was conducted utilizing the ImageJ software. The bars represent the mean ± SD. A one-way ANOVA test was conducted to assess the significant distinction among the various study groups. *** *p* < 0.001, **** *p* < 0.0001 as compared to control group. #### when comparing to ulcer rats *p* < 0.0001.

Subsequently, we conducted an examination of the expression levels pertaining to the canonical inflammatory pathway MAPK within the stomach tissues. As depicted in Figs 15 and S17, the administration of indomethacin leaded to a notable augmentation in the expression of p/t-ERK1/2 (2.01-fold), p/t-P38 (2.07-fold), and p/t-JNK (3.3-fold) relative to the control rats (*p* < 0.0001). Notably, the administration of *T*. *aphylla* extract exhibited a noteworthy suppression of the expression of p/t-ERK (1.44-fold), p/t-P38 (1.71-fold), and p/t-JNK (2.01-fold). In a similar manner, the treatment with famotidine leaded to a notable decrease in the heightened levels of p/t-ERK (1.33-fold), p/t-P38 (1.59-fold), and p/t-JNK (1.7-fold) proteins relative to the rats treated with indomethacin (*p* < 0.0001) (Fig 15).

## 4. Discussion

Herbal therapy has emerged as a popular choice for preventing and treating several diseases, particularly drug-induced gastric ulcer. Owing to their effectiveness and safety, herbal remedies have become a preferred option over authorized chemical medications. These phytomedicines contain various phytonutrients, which possess exceptional anti-inflammatory and antioxidant properties, making them valuable in the management of various disorders [85]. Additionally, *T*. *aphylla* has been found to have diverse biological importance, including anti-inflammatory, antibacterial, antifungal, treatment of periodontal diseases, anticholinesterase, analgesic, anti-diabetic, and cytotoxic activities [35].

The major goal of this study was to investigate the possible anti-ulcer activities of *T*. *aphylla* crude leaf extract in combating stomach ulcers caused by indomethacin. Additionally, the research aimed to shed light on the underlying mechanism through which these effects are exerted. Our findings unveiled that *T*. *aphylla* crude extract administration exerted a potent ameliorative potential against indomethacin-induced gastric ulcers in rats. Pre-administration of *T*. *aphylla* crude extract notably reduced the total gastric lesion area in comparison to untreated rats, which was corroborated by histological evaluation. *T*. *aphylla* crude extract impressively mitigated the damage to epithelium and necrotic lesion formation caused by indomethacin.

In previous studies, it was found that there are various flavonoids present in the crude extract of *T*. *aphylla*. There have been reports indicating that certain secondary metabolites with antioxidant properties, such as flavonoids, can help protect against the development of gastric lesions caused by ulcerogenesis and necrotic agents. These compounds might be the cause of the observed anti-ulcer properties, and their protective effect on the mucosa seems to be due, at least partially, to an increase in mucus thickness [86, 87].

NSAIDs have been observed to induce tissue oxidation, which is considered a significant detrimental impact within the gastrointestinal tract [88]. This results in gastric mucosal injuries, including hemorrhage, ulceration and erosion, through the expedited generation of hydrogen peroxide, lipid peroxidation, and protein oxidation. Antioxidants possess the potential to scavenge ROS, thereby safeguarding biological membranes against the detrimental effects of oxidative damage. GSH is a naturally occurring antioxidant that serves a crucial function in maintaining the health of mucosal tissues [89]. MDA serves as the end product of the process known as lipid peroxidation. It is commonly employed as a biomarker to assess the extent of tissue damage [90]. Depletion of antioxidant enzymes and presence of increased MDA are widely recognized as dependable biomarkers for assessing the extent of oxidative stress [90]. Our study showed that exposure to indomethacin led to a significant reduction in GSH content and increased MDA levels. The findings of the current study are compatible with previous research which confirmed the implication of oxidative stress in gastric ulcer [91–93]. On the other hand, pre-treatment with *T*. *aphylla* crude leaf extract, by contrast, resulted in significant increases in GSH and led to a reduction in MDA levels, demonstrating its antioxidant potential and confirming its gastro protective properties against the development of indomethacin-induced ulcers.

Administration of indomethacin has been implicated in exacerbating mucosal damage by triggering the release of inflammatory cytokines [16]. The emergence of-inflammatory cytokines within the gastric mucosa intensifies the inflammatory response of the gastric mucosa [94]. Indomethacin boosts an inflammatory reaction that activates macrophages to produce huge amounts of potent inflammatory cytokines, including IL-1β, IL-6, and TNF-α. These cytokines promote the infiltration of neutrophils into the inflamed area, result in the disruption of connexin and subsequent destruction of the mucosal barrier [16]. It is postulated that these inflammatory cytokines possess the capacity to induce an upsurge in the production of ROS, which can consequently contribute to the pathogenesis of gastric ulcers. Our study results concur with previous reports that support this hypothesis [95]. In contrast to the control group, the rats that were subjected to indomethacin displayed heightened levels of IL-1β, IL-6, and TNF-α [96]. Prior research has found compelling evidence of a significant rise in proinflammatory cytokines in indomethacin induced gastric ulcer emphasizing the crucial role of pro inflammatory cytokines in the development of gastric ulcer [85, 97, 98]. Pre-treatment with a crude leaf extract of *T*. *aphylla*, in contrast, exhibited inhibitory effects on the upregulation of TNF-α, IL-1 β, and IL-6 levels, and even restored these cytokines to their baseline levels. This observation highlights the anti-inflammatory properties of the extract in the context of indomethacin-induced gastric ulcer in rats. This observation aligns with the aforementioned pathological findings, which revealed reduced inflammatory responses in *T*. *aphylla* pre-treated rats with gastric ulcer. Thess anti-inflammatory properties are attributed to the high flavonoids content. Past research has indicated that antioxidants, including flavonoids, can impede the activation of NF-κB, a protein that plays a role in triggering the transcription of TNF-α, IL-1β, and IL-6 [99, 100].

The anti-gastric ulcer effect of *T*. *aphylla* crude extract was guided by a network pharmacology study where the eight identified compounds were annotated by 123 unique targets, among which 45 targets were related to peptic ulcer. The targets related to peptic ulcer conditions involved 11 targets only related to gastric ulcer, these targets are PTGS2, NOS2, MMP13, CFTR, MMP3, MMP2, MET, PLG, KDR, MMP9 and TERT. The PTGS2 gene was found to be the most represented gene by seven compounds related to gastric ulcer. The present results agreed with previously published data that refer the role of PTGS2 (known as cyclooxygenase-2) to have a protective role against gastric ulcer, their inhibitors delay the gastric ulcer healing, The enhanced level of COX-2 mRNA and protein near the ulcer border is spatially and temporally associated with increased the proliferation of epithelial cell and heightened expression of growth factors. Additionally, mounting evidence suggests that exposure to hazardous stimuli or ischaemia-reperfusion destruction increases the production of COX-2 mRNA and protein in the stomach mucosa [101]. The top signaling pathway was the EGFR tyrosine kinase inhibitor resistance pathway. This pathway is related to different cascades including MAPK signaling pathway, PI3K-AKT signaling pathway and mTOR signaling pathway. The MAPK signaling pathway was proved to be related to gastric ulcer inhibition, so the *in vivo* animal study focused on the MAPK signaling pathway genes.

The MAPKs pathway, a crucial component in cellular signaling, plays a pivotal role in governing a wide array of biological processes. MAPKs constitute a group of enzymes primarily responsible for orchestrating the cellular reaction to extrinsic stress stimuli and pro-inflammatory cytokines. The primary subfamilies of MAPKs include ERKs, p38 kinases, and c-JNKs. These subfamilies have been observed to potentially mitigate the extent of gastric injury. They exhibit a pivotal function in regulating the expression of a diverse range of inflammatory mediators [102]. Recent scientific investigations have revealed that the induction of gastric injury by indomethacin is closely linked to the activation of MAPKs. Consequently, the inhibition of MAPKs is expected to present a promising avenue for the therapeutic intervention of stomach injury [103]. It is well-established that this pathway is activated by upstream stimulators, including TNF-α and IL-6, which subsequently initiate a cascade of events leading to the production of downstream products. Furthermore, it has been demonstrated that the upregulation of TNF-α and IL-6 leads to an augmentation in the phosphorylation of ERK, p38, and c-JNK. These protein kinases are of utmost importance in the progression of tissue damage [104, 105]. Prior research has indicated that the activation of p38 MAPK is involved in the impairment of the gastric epithelial barrier induced by NSAIDs [106, 107]. Additionally, it has been demonstrated that the inhibition of MAPKs can augment the functionality of the epithelial barrier [108]. Furthermore, the ERK pathway plays a crucial role in the regulation of gastric acid secretion and the process of wound healing. Studies have shown that inhibiting the phosphorylation of ERK can effectively alleviate hyperacidity and decrease the expression of matrix metalloproteinases, ultimately facilitating the recovery of gastric injuries [15, 109]. These findings provide additional evidence to substantiate our hypothesis that the beneficial effect of *T*. *aphylla* to protect against indomethacin-induced gastrointestinal injury is associated with the downregulation of phosphorylated ERK, p38, and JNK. In a previous study, it was found that the increase in neutrophil infiltration caused by aspirin-induced gastric ulcer was mitigated by kaempferol treatment. This led to a decrease in the levels of JNK and p38 [110]. In addition, flavonoids were found to decrease the levels of pro-inflammatory cytokines through the MAPK pathway in mice with ethanol-induced acute gastric ulcers [100, 111].

The notable functionality of *T*. *aphylla* could potentially be ascribed to its elevated concentrations of flavonoids, which serve to maintain the gastric GSH level by functioning as scavengers of free radicals in lieu of GSH, thereby impeding lipid peroxidation. Additionally, flavonoids exert their activity through numerous possible mechanisms of action such as cytoprotectors by increasing the mucus and antioxidants preventing lipid peroxidation. They also may have immunoregulatory function by reducing the proinflammatory cytokines and increasing the anti-inflammatory ones. Moreover, their anti-secretory properties are achieved by reducing the levels of H^+^ secretion and performing anti-*H*.*-pylori* properties. Thus, they can be utilized as beneficial complementary drugs and dietary supplements to counteract the development of gastric ulcer and its incidences of recurrence and contribute mainly to the traditional cure of ulcerative lesions.

Our current study has revealed the potential therapeutic effect of *T*. *aphy*lla leaves as gastroprotective medication via down regulation of inflammatory cytokines, restore pH balance, reconstruct the mucus layer, and prevent epithelial cell loss in cases of indomethacin-induced gastrointestinal injury. This effect is mediated in part via modulating MAPKs signaling pathways. On the other hand, this study has certain limitations that should be acknowledged. First and foremost, further research and future investigations are necessary to enhance our understanding of potential alternative molecular mechanisms through which *T*. *aphylla* leaf extract could exert its gastroprotective effects against indomethacin-induced gastric ulcers. Additionally, the absence of human studies, notably randomized controlled trials, hinders the availability of additional evidence regarding the extract’s effectiveness and safety in a clinical environment.

## 5. Conclusion

In the present study, a phytochemical investigation of *T*. *aphylla* crude leaf extract has resulted in the isolation and identification of eight flavonoidal compounds, three of them were characterized as flavonoidal glycosides. Excitingly, our study has revealed the remarkable potential of *T*. *aphylla* crude extract in significantly ameliorating gastric ulcers induced by indomethacin administration. Our findings provide compelling evidence that the therapeutic efficacy of the extract can be attributed to its ability to downregulate the MAPK signaling pathway, and to its potent antioxidant effects, without compromising gastric acidity. In light of these results, *T*. *aphylla* leaf extract offers a promising and effective therapy for the treatment of inflammation and NSAID-induced gastric ulcers, while also providing a safer alternative to traditional antisecretory drugs with their notorious side effects.

## Supporting information

S1 Table Gene ontology analysis in form of biological process, cellular components and molecular functions. (JPG)

S1 Fig ^1^H NMR spectrum of compound 1 measured in CDCl_3_ at 400 MHz. (JPG)

S2 Fig ^1^H NMR spectrum of compound 2 measured in CDCl_3_ at 400 MHz. (JPG)

S3 Fig ^1^H NMR spectrum of compound 3 measured in CDCl_3_ at 400 MHz. (JPG)

S4 Fig ^1^H NMR spectrum of compound 4 measured in CDCl_3_ at 400 MHz. (JPG)

S5 Fig ^1^H NMR spectrum of compound 5 measured in CDCl_3_ at 400 MHz. (JPG)

S6 Fig ^1^H NMR spectrum of compound 6 measured in DMSO-d_6_ at 400 MHz. (JPG)

S7 Fig DEPT-Q NMR spectrum of compound 6 measured in DMSO-d_6_ at 100 MHz. (JPG)

S8 Fig ^1^H NMR spectrum of compound 7 measured in CD_3_OD at 400 MHz. (JPG)

S9 Fig DEPT-Q NMR spectrum of compound 7 measured in CD_3_OD at 100 MHz. (JPG)

S10 Fig ^1^H NMR spectrum of compound 8 measured in CD_3_OD at 400 MHz. (JPG)

S11 Fig DEPT-Q NMR spectrum of compound 8 measured in CD_3_OD at 100 MHz. (JPG)

S12 Fig Plant—cpd network; yellow oval shape is the plant name; oval pink shapes are the identified compounds. (JPG)

S13 Fig Cpds—target network; a network connects the identified compounds with their corresponding targets. The diamond violet shapes represent the identified compounds and the yellow oval shapes represent the targets.
(JPG)

S14 Fig Cpds—gastric ulcer network; yellow rectangle is the gastric ulcer condition, blue rectangles represent targets related to gastric ulcer. (JPG)

S15 Fig Gastric ulcer targets—cpds network; pink rectangles represent the compounds identified from *T*. *aphylla*, yellow rectangles represent the gastric ulcer targets. (JPG)

S16 Fig Gene ontology and enrichment analysis in terms of biological process, cellular component and molecular function. (JPG)

S17 Fig Representative immune blots of A: t-ERK1/2, B: p-ERK1/2, C:p & t-MAPK P38, D: t & p-JNK in all the study groups.
(JPG)
