# Supplementary figures and images for: Tamarix aphylla derived metabolites ameliorate indomethacin-induced gastric ulcers in rats by modulating the MAPK signaling pathway, alleviating oxidative stress and inflammation: In vivo study supported by pharmacological network analysis
Source: PLoS One. 2024 May 10;19(5):e0302015. doi: 10.1371/journal.pone.0302015 (PMC11086843; doi:10.1371/journal.pone.0302015)

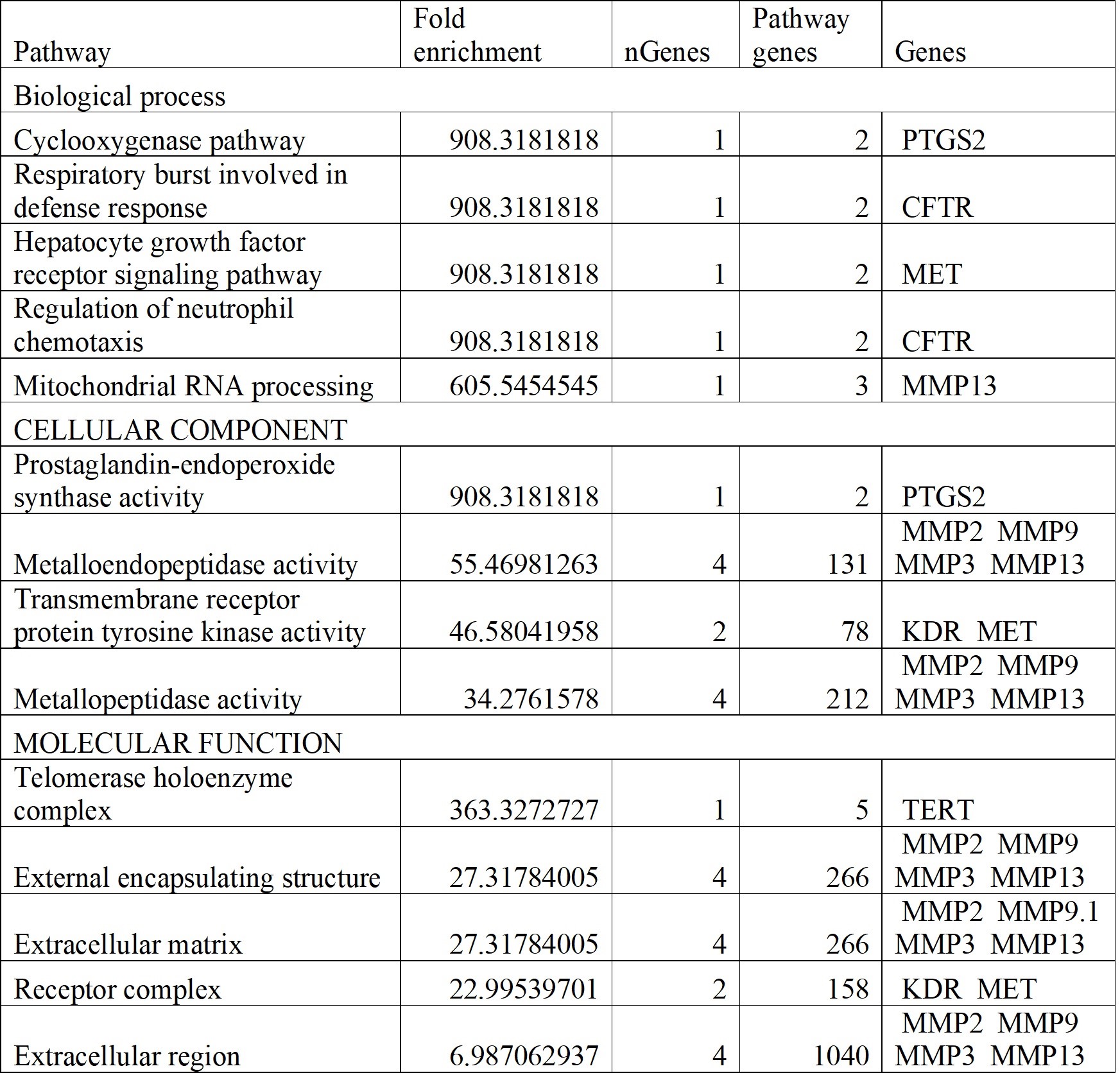

Supplement: S1 Table — (JPG) [file pone.0302015.s001.jpg]

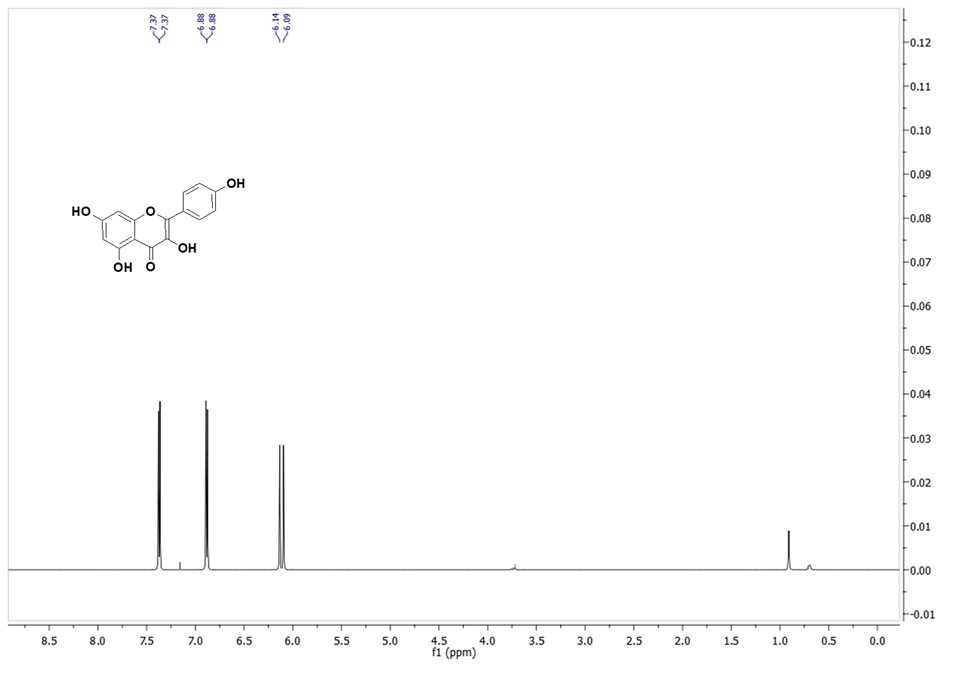

Supplement: S1 Fig — (JPG) [file pone.0302015.s002.jpg]

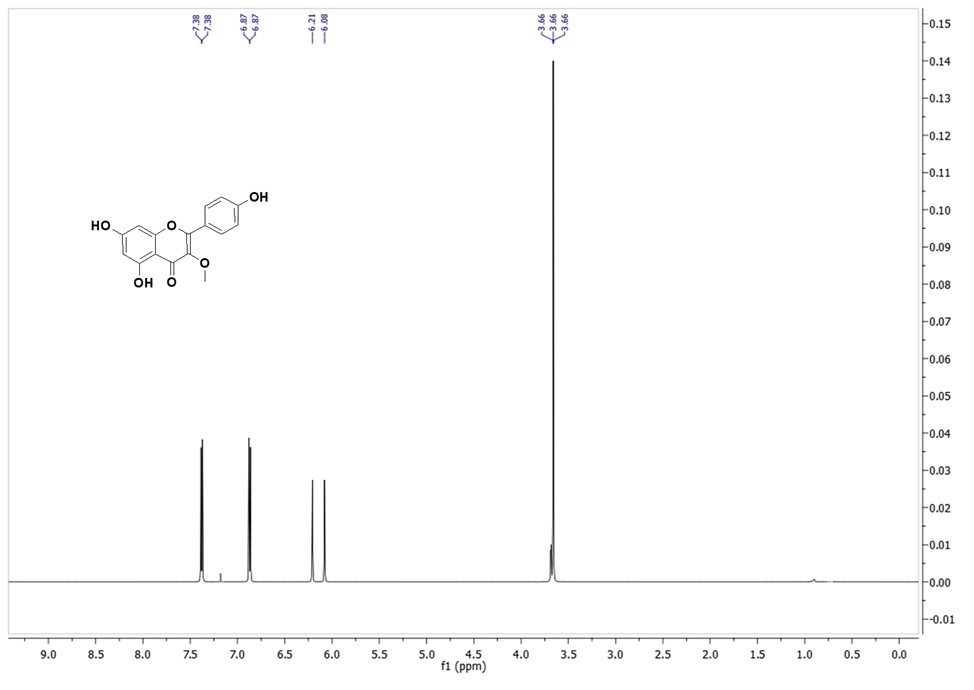

Supplement: S2 Fig — (JPG) [file pone.0302015.s003.jpg]

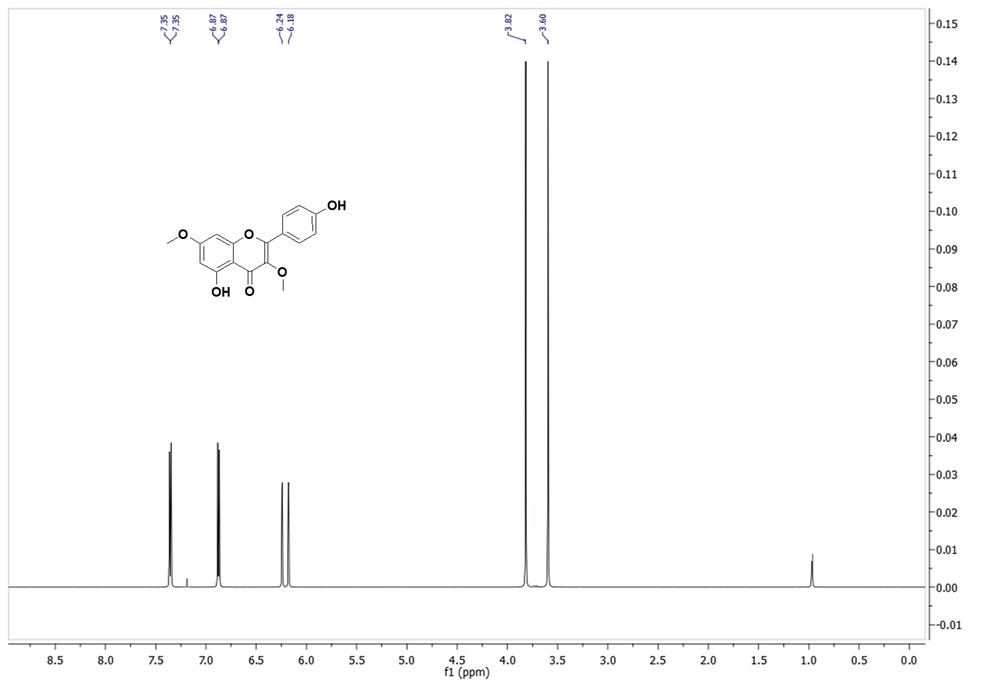

Supplement: S3 Fig — (JPG) [file pone.0302015.s004.jpg]

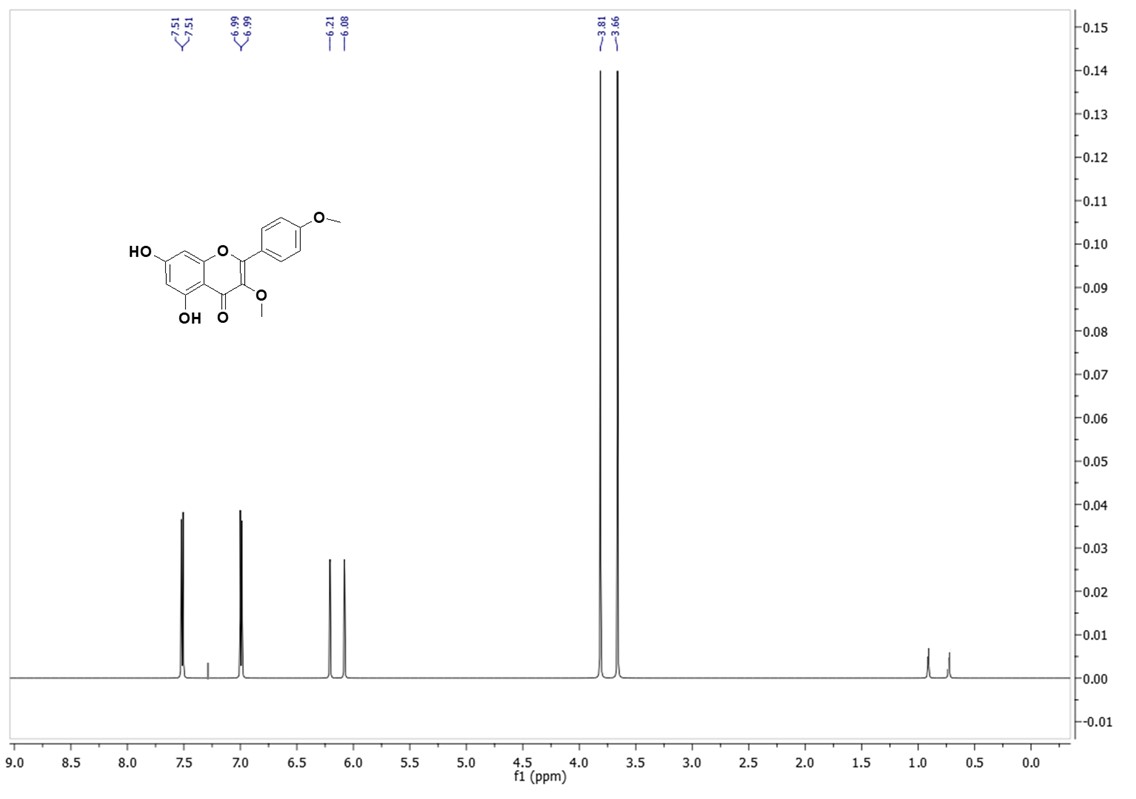

Supplement: S4 Fig — (JPG) [file pone.0302015.s005.jpg]

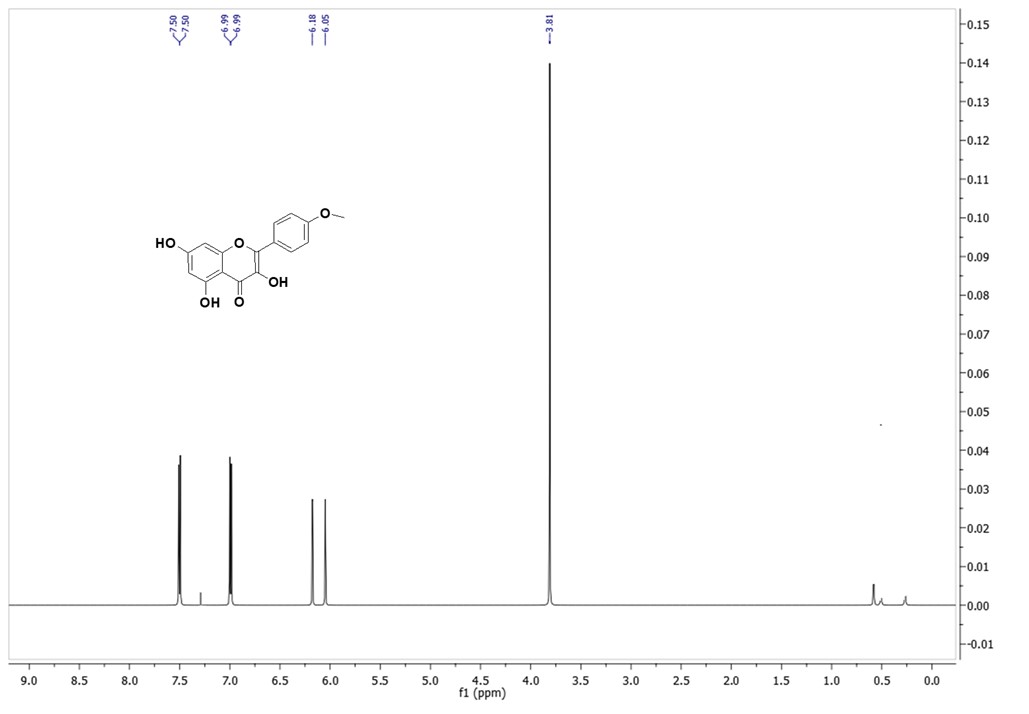

Supplement: S5 Fig — (JPG) [file pone.0302015.s006.jpg]

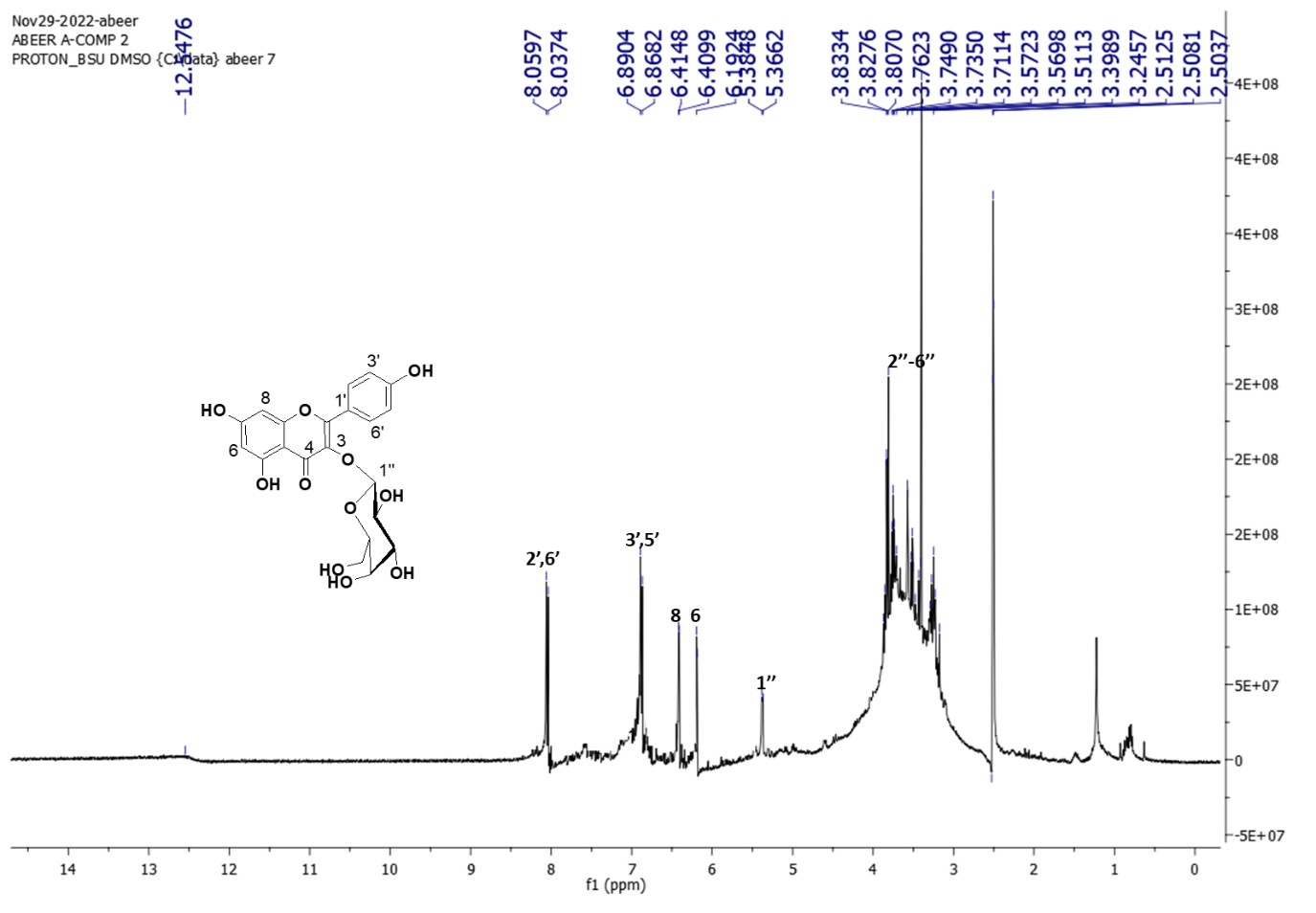

Supplement: S6 Fig — (JPG) [file pone.0302015.s007.jpg]

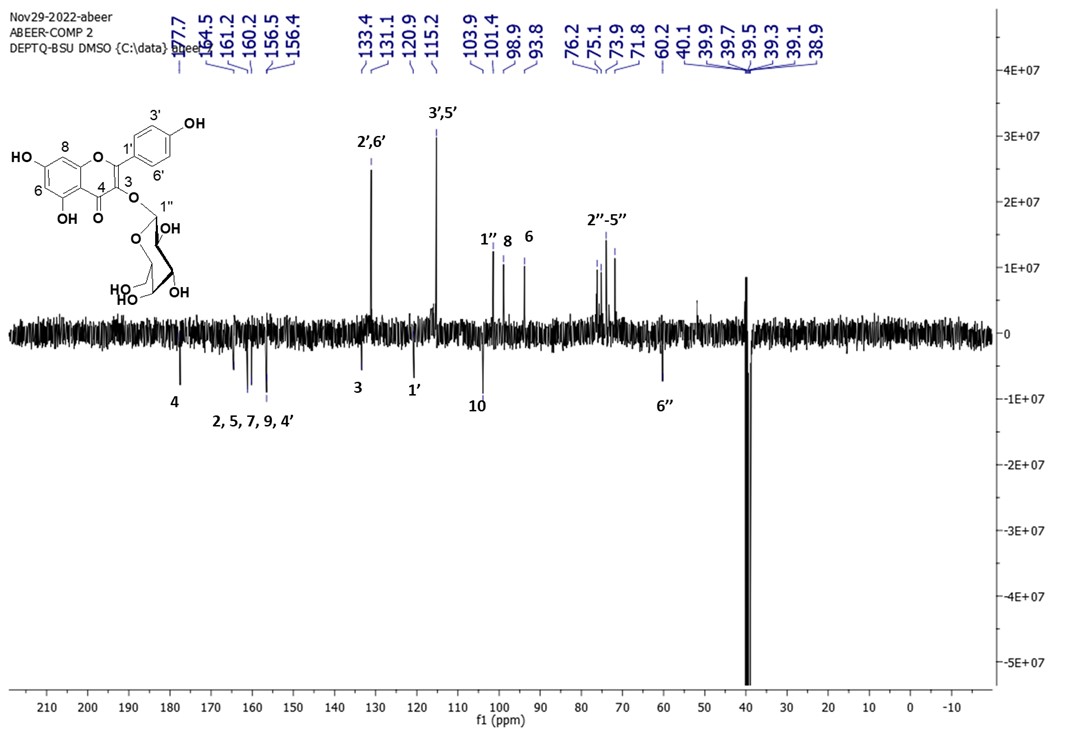

Supplement: S7 Fig — (JPG) [file pone.0302015.s008.jpg]

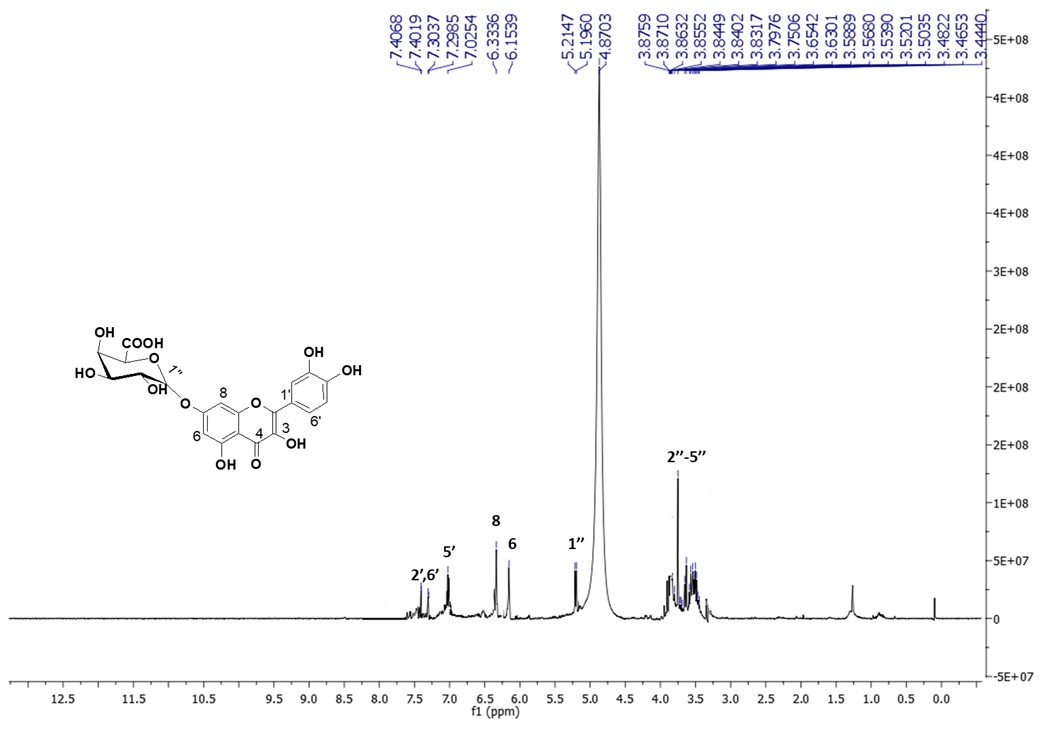

Supplement: S8 Fig — (JPG) [file pone.0302015.s009.jpg]

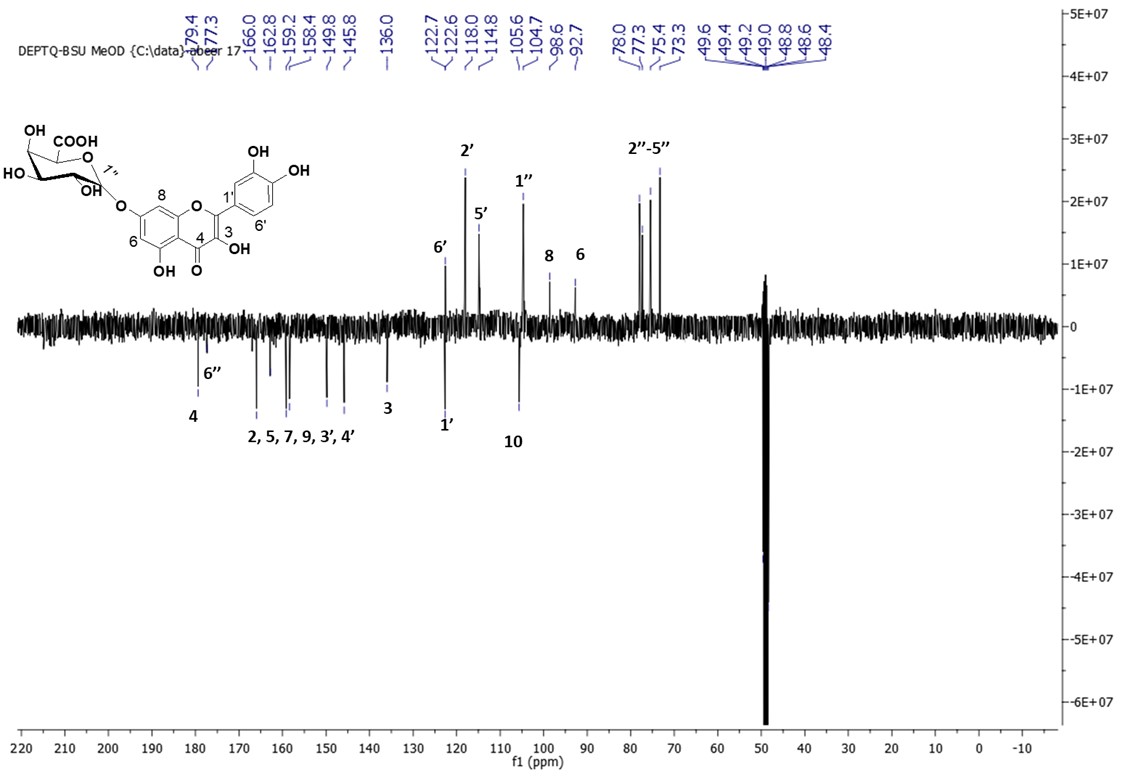

Supplement: S9 Fig — (JPG) [file pone.0302015.s010.jpg]

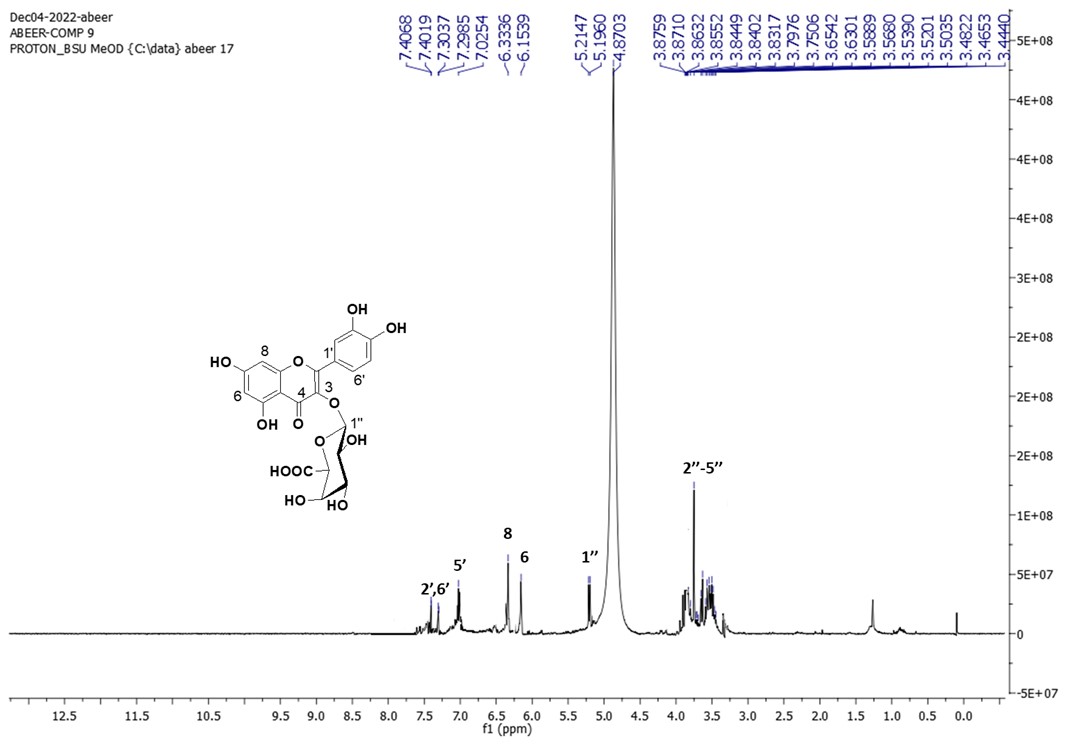

Supplement: S10 Fig — (JPG) [file pone.0302015.s011.jpg]

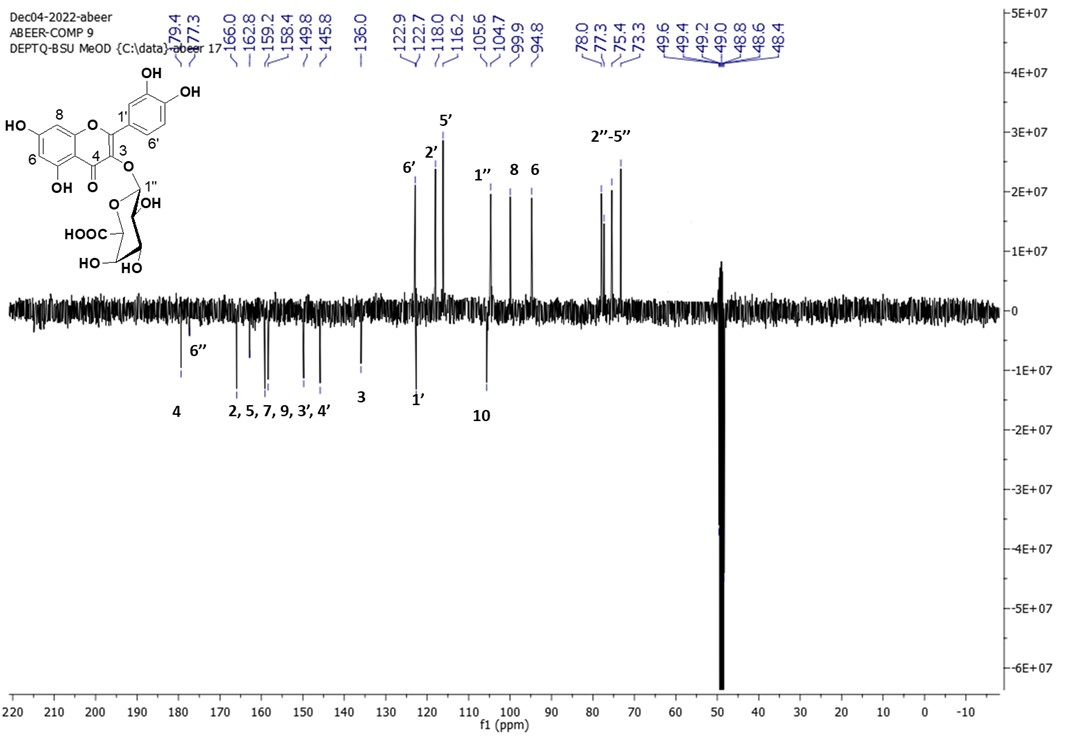

Supplement: S11 Fig — (JPG) [file pone.0302015.s012.jpg]

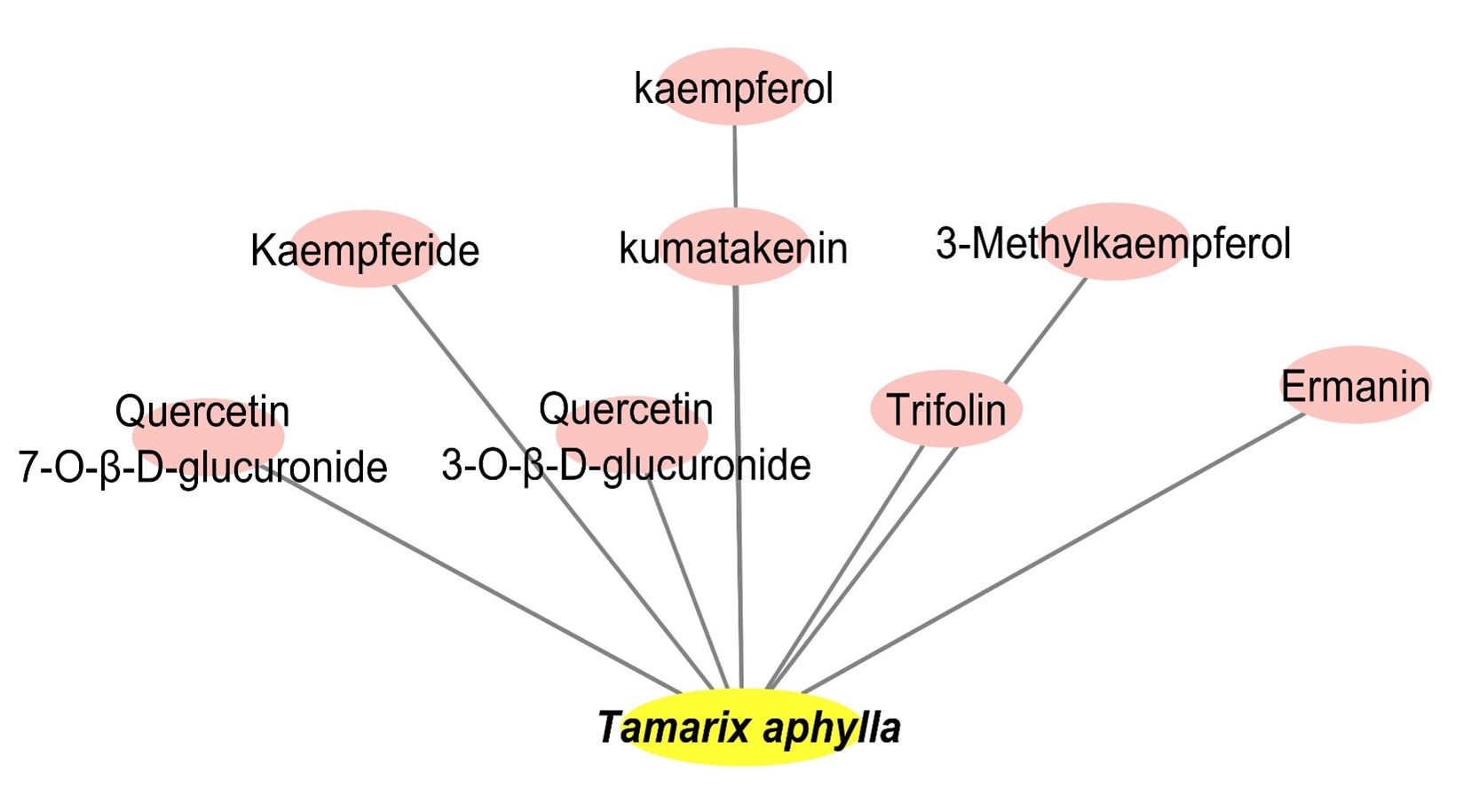

Supplement: S12 Fig — (JPG) [file pone.0302015.s013.jpg]

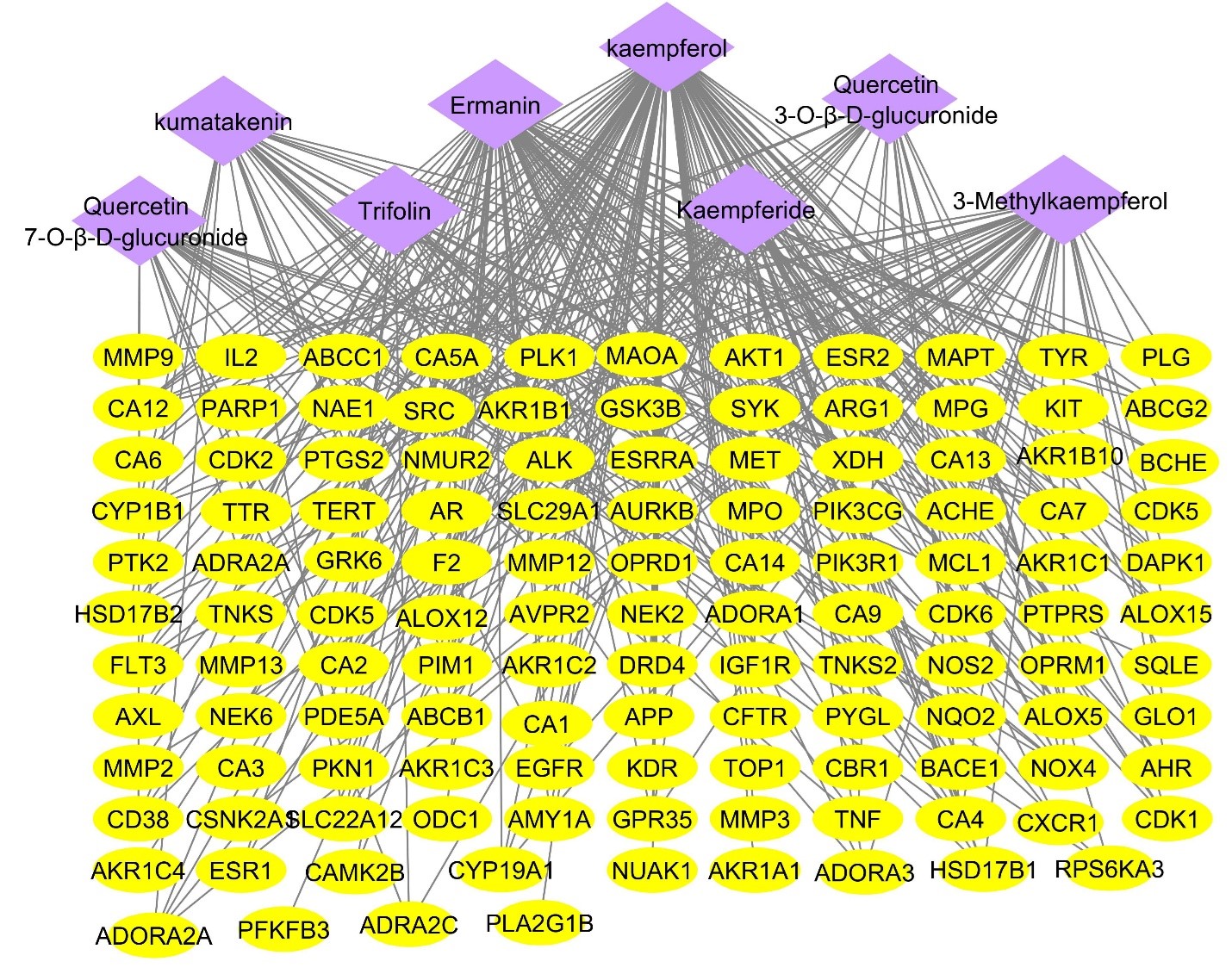

Supplement: S13 Fig — The diamond violet shapes represent the identified compounds and the yellow oval shapes represent the targets. (JPG) [file pone.0302015.s014.jpg]

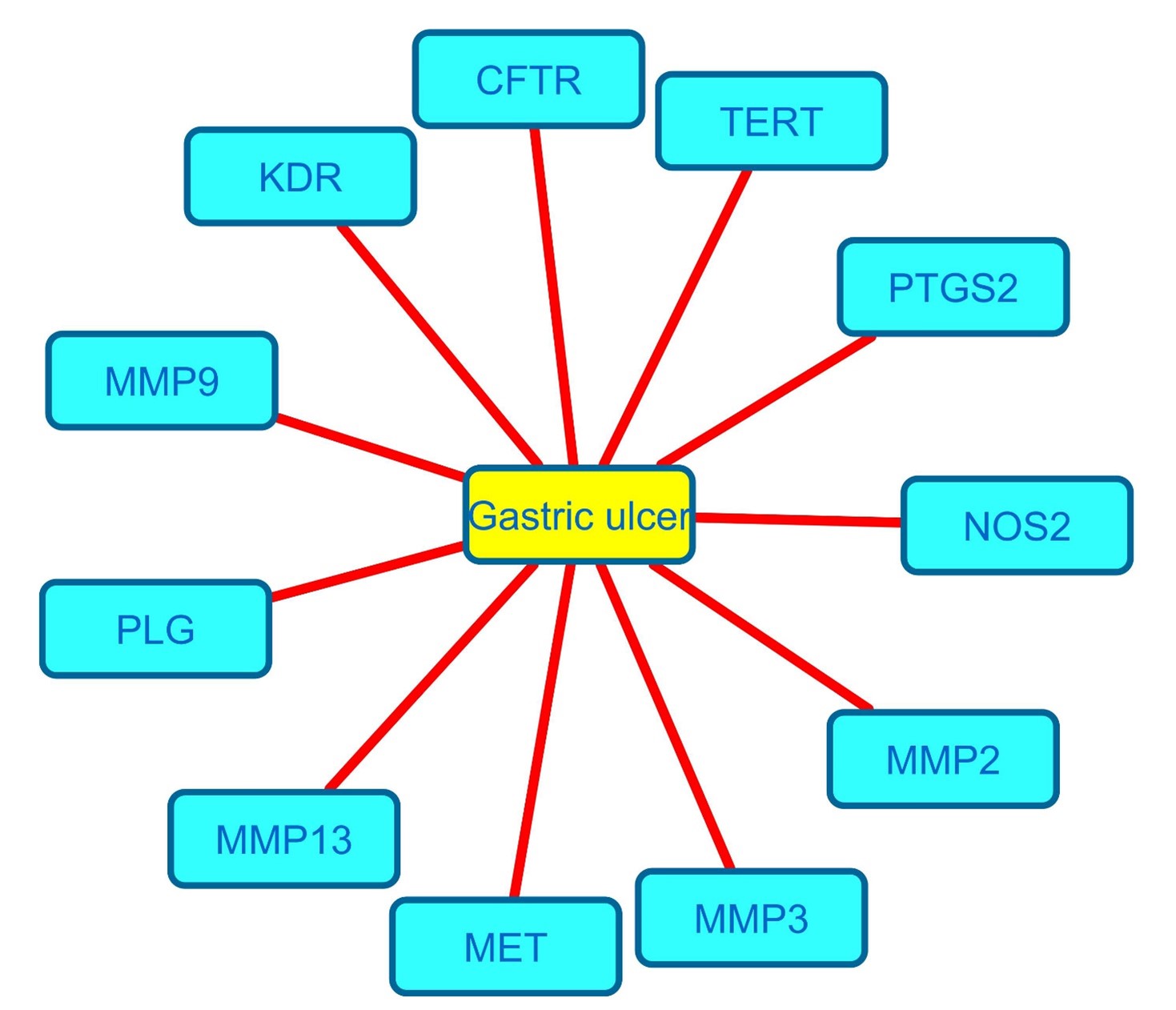

Supplement: S14 Fig — (JPG) [file pone.0302015.s015.jpg]

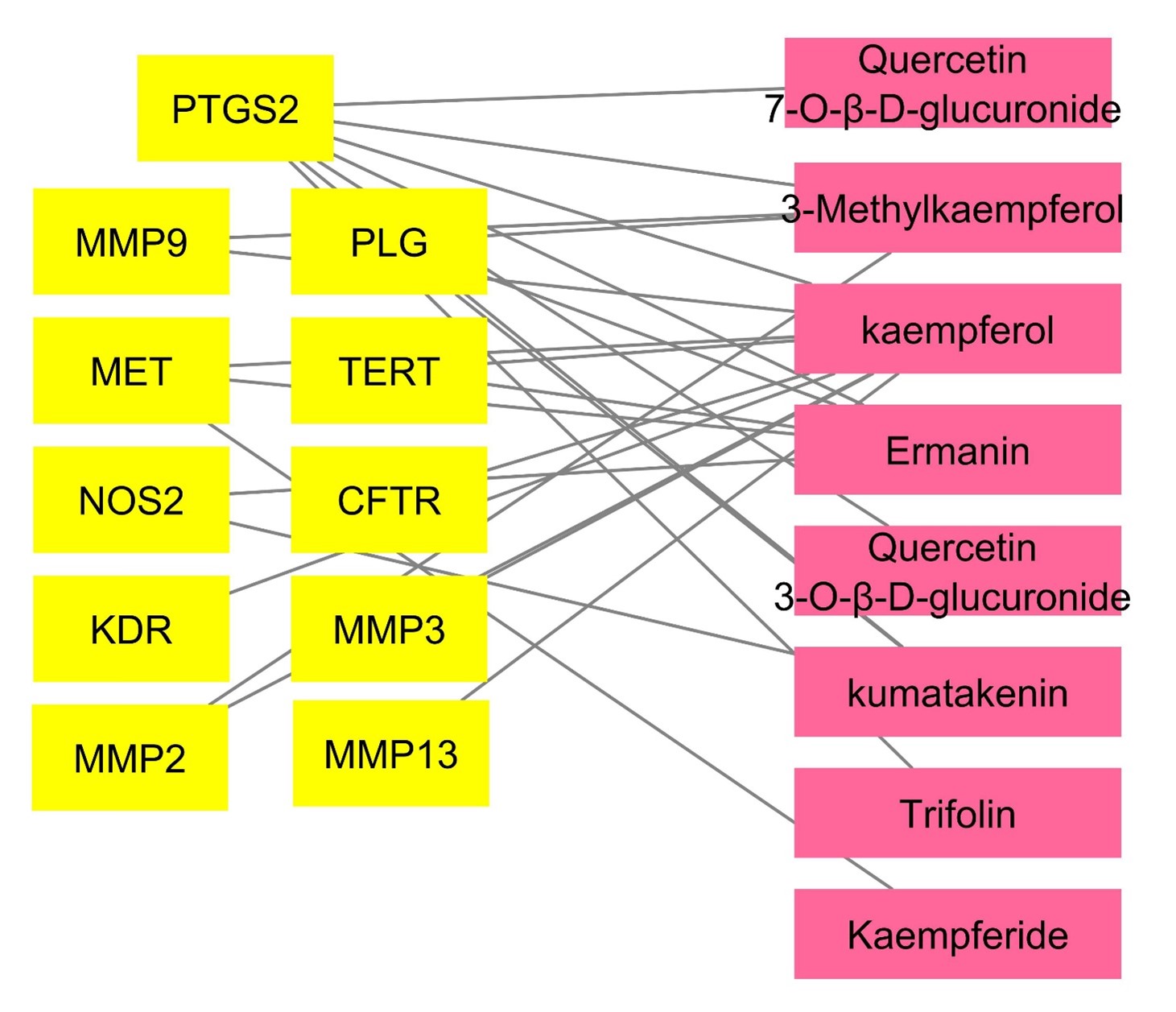

Supplement: S15 Fig — (JPG) [file pone.0302015.s016.jpg]

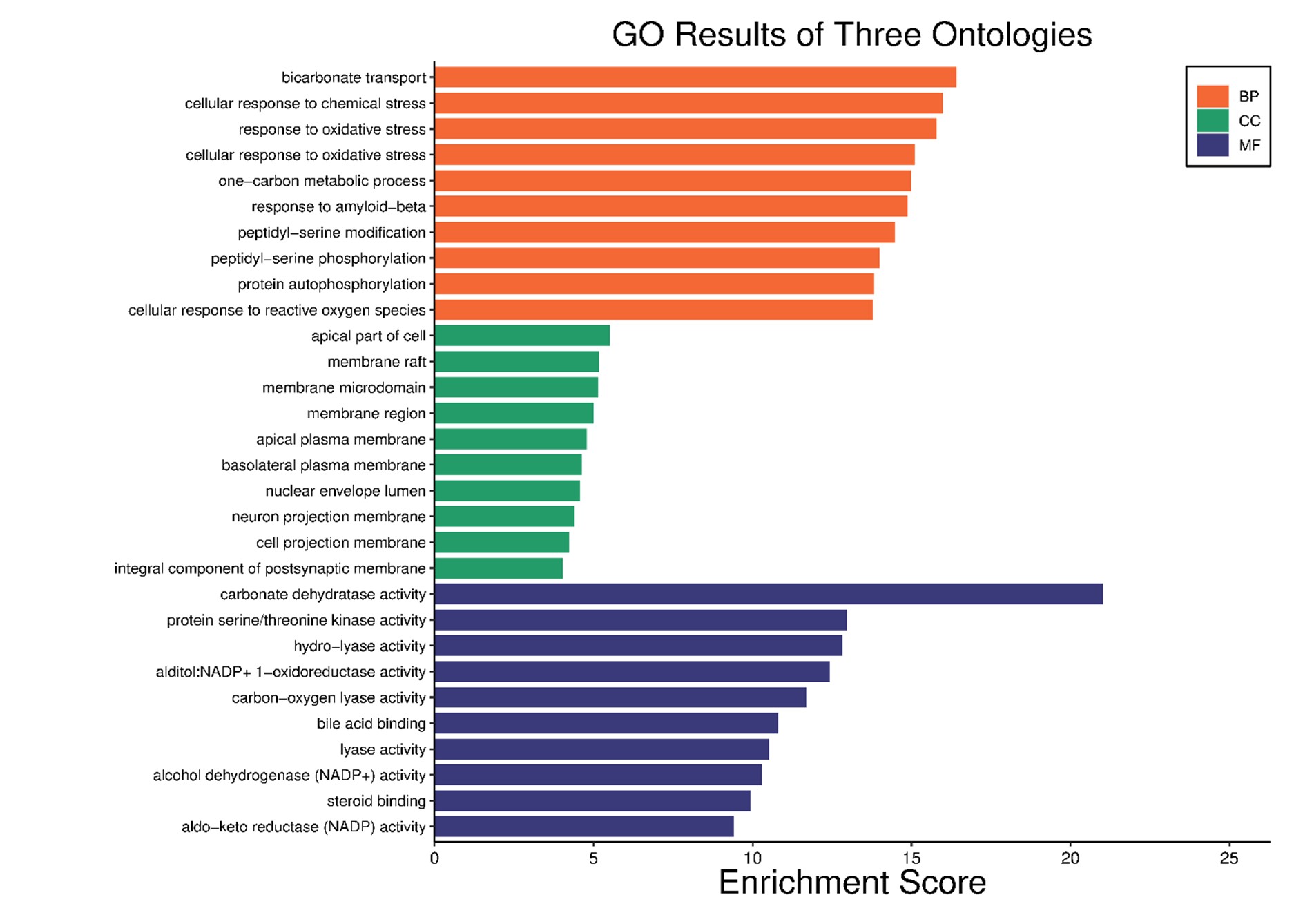

Supplement: S16 Fig — (JPG) [file pone.0302015.s017.jpg]

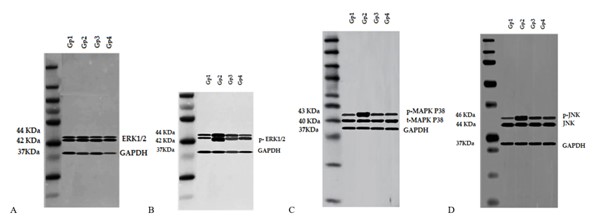

Supplement: S17 Fig — Representative immune blots of A: t-ERK1/2, B: p-ERK1/2, C:p & t-MAPK P38, D: t & p-JNK in all the study groups. (JPG) [file pone.0302015.s018.jpg]
